# Exploring the antimicrobial potential of lactobacilli against early-stage and mature biofilms of *Staphylococcus aureus* and *Pseudomonas aeruginosa*

**DOI:** 10.3389/fchem.2025.1425666

**Published:** 2025-03-21

**Authors:** Niharika Singh, Rohini Devidas Gulhane, Anamika Singh, Maitri Goel, Pudke Payal Udelal, Vikas Sangwan, Manvesh Kumar Sihag, Gunjan Goel, Harsh Panwar, Anil Kumar Puniya

**Affiliations:** ^**1**^ Department of Dairy Microbiology, College of Dairy and Food Science Technology, Guru Angad Dev Veterinary and Animal Sciences University (GADVASU), Ludhiana, Punjab, India; ^2^ Department of Biotechnology, VSB Engineering College, Karur, Tamil Nadu, India; ^3^ Department of Dairy Chemistry, College of Dairy and Food Science Technology, Guru Angad Dev Veterinary and Animal Sciences University (GADVASU), Ludhiana, Punjab, India; ^4^ Department of Microbiology, School of Interdisciplinary and Applied Science, Central University of Haryana, Mahendergarh, India; ^5^ Dairy Microbiology Division, ICAR-National Dairy Research Institute, Karnal, Haryana, India

**Keywords:** lactic acid bacteria, antimicrobial, probiotics, cell-free supernatant, biofilm, foodborne pathogens, *Staphylococcus aureus*, *Pseudomonas aeruginosa*

## Abstract

Bacterial biofilms are dynamic, complex, and very adaptive, and they can cause health problems in both humans and animals while also posing a serious threat to various industries. This study explores the potential of cell-free preparations of lactobacilli isolated from breast milk (HM; n = 11) and infant fecal (IF; n = 15) samples to impact the growth of *Staphylococcus aureus* and *Pseudomonas aeruginosa* biofilms. The anti-biofilm activity of three distinct cell-free preparations, namely, untreated cell-free supernatant (CFS), pH-neutralized CFS (N-CFS), and heat-treated CFS (H-CFS), was examined against both early-stage and mature biofilms. The post-incubation strategy examined the impact on mature biofilms, while the co-incubation treatment assessed the impact of CFS on adhesion and initial colonization. Compared to post-incubation treatment (HM3, 67.12%), the CFSs exhibited greater inhibitory activity during co-incubation (IF9, 85.19%). Based on the findings, untreated CFS exhibited the most promising biofilm inactivation, although its activity was not completely lost upon pH neutralization and heat treatment. Treatment with H-CFSs and N-CFSs moderately reduced the population of *S. aureus* and *P. aeruginosa* bacterial cells within the biofilm by 40%–60%. Microscopic observations showed that after CFS treatment, the integrity of the biofilm conformation was disrupted. According to principal component analysis (PCA) (significance level at *p* < 0.05), the most promising anti-biofilm activity against both test pathogens was found in the CFS of *Lacticaseibacillus paracasei* HM1.

## 1 Introduction

A bacterial biofilm consists of organized bacteria surrounded by a self-produced extracellular matrix composed of proteins, DNA, and polysaccharides. This matrix provides a protective environment for the bacteria, fostering collaboration and communication among them and enabling the community to respond collectively to changes in their surroundings. Biofilm development involves different phases, *viz*., the adhesion of planktonic cells to the surface (adhesion phase), cell growth and aggregation (initial colonization phase), production of extracellular components, the eventual development of a mature biofilm matrix (maturation phase), and cellular detachment or dispersal (biofilm dispersal) (Drago et al., 2024; Schilcher and Horswill, 2020; Singh et al., 2018). Biofilms are a significant challenge in both the food industry and healthcare settings. They can harbor foodborne pathogens, leading to spoilage and health risks for consumers. In hospitals, they persist on medical device surfaces and patient tissues, causing persistent infections (Li et al., 2023; Liu et al., 2023). Biofilm formation is an important virulence attribute of pathogens including *Staphylococcus aureus* and *Pseudomonas aeruginosa* (Schilcher and Horswill, 2020). The property of pathogenic and spoilage microorganisms to form biofilm represents a challenge to the food industry since it leads to food contamination, infections, disease transmission, and economic losses (Olanbiwoninu and Popoola, 2023). It is now well-established that the persistence of pathogenic bacteria on food processing equipment leads to foodborne outbreaks, particularly those involving *S. aureus* and *P. aeruginosa*, which rank among the top four microbes isolated from healthcare-associated infections (HAIs) in the European Union (EU) (Zarb et al., 2012). Based on statistics published by the European Food Safety Authority and the European Centre for Disease Control (European Food Safety Authority and European Centre for Disease Prevention and Control, 2016), bacterial toxins were the third most common cause of foodborne illnesses in the European Union in 2015, accounting for 19.5% of all foodborne outbreaks. In 2015, 434 foodborne outbreaks caused by staphylococcal toxins were reported by 16 Member States (MSs), accounting for 9.9% of all outbreaks and a modest increase over the previous year. The National Healthcare Safety Network reported that *S. aureus* is associated with 15% and *P. aeruginosa* with 8% of total HAIs in the United States (Hidron et al., 2008; Sievert et al., 2013). *S. aureus* colonizes both the abiotic surfaces and human tissues and constitutes one of the most important causative agents of nosocomial infections related to implanted medical devices (European Centre for Disease Prevention and Control et al., 2013; Otto, 2013). According to Cross et al. (1983), it is one of the top three causes of opportunistic infections in humans, affecting approximately 2 million patients annually and causing nearly 9,000 deaths. Owing to its ability to secrete extracellular enzymes, *P. aeruginosa* is frequently found as a spoilage bacterium, especially in foods with high water content and high nutritional value. *P. aeruginosa* also ranks as the sixth most common nosocomial pathogen overall and the second most common pathogen in ventilator-associated pneumonia in United States hospitals (Weiner et al., 2016).

Bacterial cells in biofilms also display resistance toward antimicrobial agents and host immune defense systems compared to their planktonic counterparts; this resistance results from various synergistic mechanisms that protect the bacteria within the biofilm (Usman et al., 2024). Currently, food safety has been the greatest concern of consumers, the food industry, and public health authorities; consequently, the development of novel perceptions and the discovery of anti-biofilm agents are expected to be promising strategies.

Conventional approaches, principally based on antimicrobial agents, may not achieve maximal efficacy against resident microbes in the complex biofilm microenvironment. In recent years, several controls and supplementary anti-biofilm strategies, *viz*., antimicrobial peptide coating, bacteriophages, enzymes, quorum quenchers, nanoparticles, and essential oils, have been evaluated (Nag et al., 2024; Glizniewicz et al., 2024; Parga et al., 2023; Song et al., 2023; Si et al., 2024; Ersanli et al., 2023).

In addition to antimicrobials, microbial metabolites, particularly those produced by lactic acid bacteria (LAB), are emerging as a promising control strategy to control biofilm-forming pathogens (Miquel et al., 2016; Simoes et al., 2010). LAB with generally recognized as safe (GRAS) status are commonly used as probiotics for mitigating inflammatory responses and metabolic disorders, such as inflammatory bowel syndrome (Chauhan et al., 2014; Elshaghabee et al., 2017; Panwar et al., 2013; Thakur et al., 2016), and their potential to prevent or disrupt biofilm formation by pathogenic bacteria could be promising in the context of food safety. The strain-specific anti-biofilm-forming abilities of LAB against several pathogens have been well-reported in different studies (Perez-Ibarreche et al., 2016; Singh et al., 2018). The strain-specific activities of LAB imply that more investigations are required to elucidate the efficacy and the underlying mechanisms of different LAB strains from varied sources.

LAB naturally present in the healthy human infant gut and various fermented foods are being widely explored as potential probiotics. The composition LAB residing in the infant’s gut is reported to be influenced by the feeding regimen, such as mother’s milk (breast milk) or formula milk. Mother’s milk has been shown to share microbial strains and other biotherapeutics with the developing infant gut (Singh et al., 2021); nevertheless, its potential as a source for isolating LAB strains and studying their anti-biofilm abilities remains unexplored. In this study, we evaluated the antimicrobial activity of cell-free supernatant (CFS) preparations from LAB strains isolated from breast milk and healthy human infant fecal samples against *S. aureus* and *P. aeruginosa* at different stages. Microscopic examinations were also carried out to decipher the changes in the biofilm structure induced by CFS preparations.

## 2 Materials and methods

### 2.1 Bacterial strains

A total of twenty-six LAB strains isolated from breast milk (HM; n = 11) and infant fecal samples (IF; n = 15) (Table 1), along with two reference probiotic strains, namely, *Lacticaseibacillus rhamnosus* GG (ATCC 53103) and *Lacticaseibacillus casei* (ATCC 393), were evaluated for their anti-biofilm activity against *S. aureus* (MTCC 96) and *P. aeruginosa* (MTCC 741). The isolates were earlier identified and characterized at the species level based on the morphological and biochemical methods and using genus-specific PCR and 16S rRNA-based sequencing (Panwar et al., 2016). The purity of cultures was occasionally ascertained by MALDI-TOF-MS-based identification at the central facility available at GADVASU. All the bacterial strains, available as glycerol stock (−80°C) at the repository of the Department of Dairy Microbiology, were revived, sub-cultured, and tested for purity. LAB strains were propagated and maintained in De Man-Rogosa-Sharpe (MRS) broth (HiMedia Laboratories Pvt. Limited, India), while the brain heart infusion (BHI) broth (HiMedia Laboratories Pvt. Limited, India) was used for culturing pathogenic strains. All the strains were sub-cultured thrice prior to the experiment.

**TABLE 1:** | List of lactic acid bacteria strains used in the study.

Isolates	Identity
HM1	*Lacticaseibacillus paracasei*
HM2	*Lactiplantibacillus plantarum*
HM3	*Lactobacillus sp.*
HM6	*Lactiplantibacillus pentosus*
HM7	*Lactiplantibacillus plantarum*
HM8	*Lactiplantibacillus plantarum*
HM9	*Lactiplantibacillus plantarum*
HM10	*Lactiplantibacillus plantarum*
HM11	*Lactiplantibacillus plantarum*
HM12	*Lactobacillus sp.*
HM13	*Lactiplantibacillus pentosus*
IF1	*Ligilactobacillus salivarius*
IF2	*Ligilactobacillus salivarius*
IF3	*Limosilactobacillus fermentum*
IF4	*Limosilactobacillus fermentum*
IF5	*Limosilactobacillus fermentum*
IF6	*Limosilactobacillus fermentum*
IF7	*Enterococcus faecalis*
IF8	*Limosilactobacillus mucosae*
IF9	*Lacticaseibacillus rhamnosus*
IF10	*Limosilactobacillus fermentum*
IF11	*Limosilactobacillus fermentum*
IF12	*Limosilactobacillus fermentum*
IF13	*Limosilactobacillus fermentum*
IF14	*Limosilactobacillus fermentum*
IF15	*Limosilactobacillus fermentum*

### 2.2 Preparation of bacterial cell-free supernatants

LAB strains from breast milk and infant fecal origin were inoculated into MRS broth and incubated overnight at 37°C. Following incubation, cell-free supernatants were prepared by centrifuging (at 5,000 × g for 5 min) the spent cultures and filtering through 0.22-μM cellulose membrane filters (Millipore, United States), either before or after pH neutralization to generate untreated CFS and pH-neutralized N-CFS, respectively. pH neutralization eliminates organic acids and end products of metabolism that may contribute to biofilm inhibition (Wasfi et al., 2018). Furthermore, the untreated CFS was treated at 100°C for 5 min to obtain heat-treated (H-CFS) preparation (Ouali et al., 2014). Heat treatment determines the heat stability of the inhibitory component while eliminating biofilm inhibition induced by proteinaceous metabolites, such as bacteriocins and non-bacteriocin-like lytic proteins (Mariam et al., 2014). Cell-free preparations, *viz*., untreated CFS, N-CFS, and H-CFS, thus obtained were stored at −80°C until further use.

### 2.3 Effect of cell-free supernatants from LAB on biofilm inhibition

**FIGURE 1:**
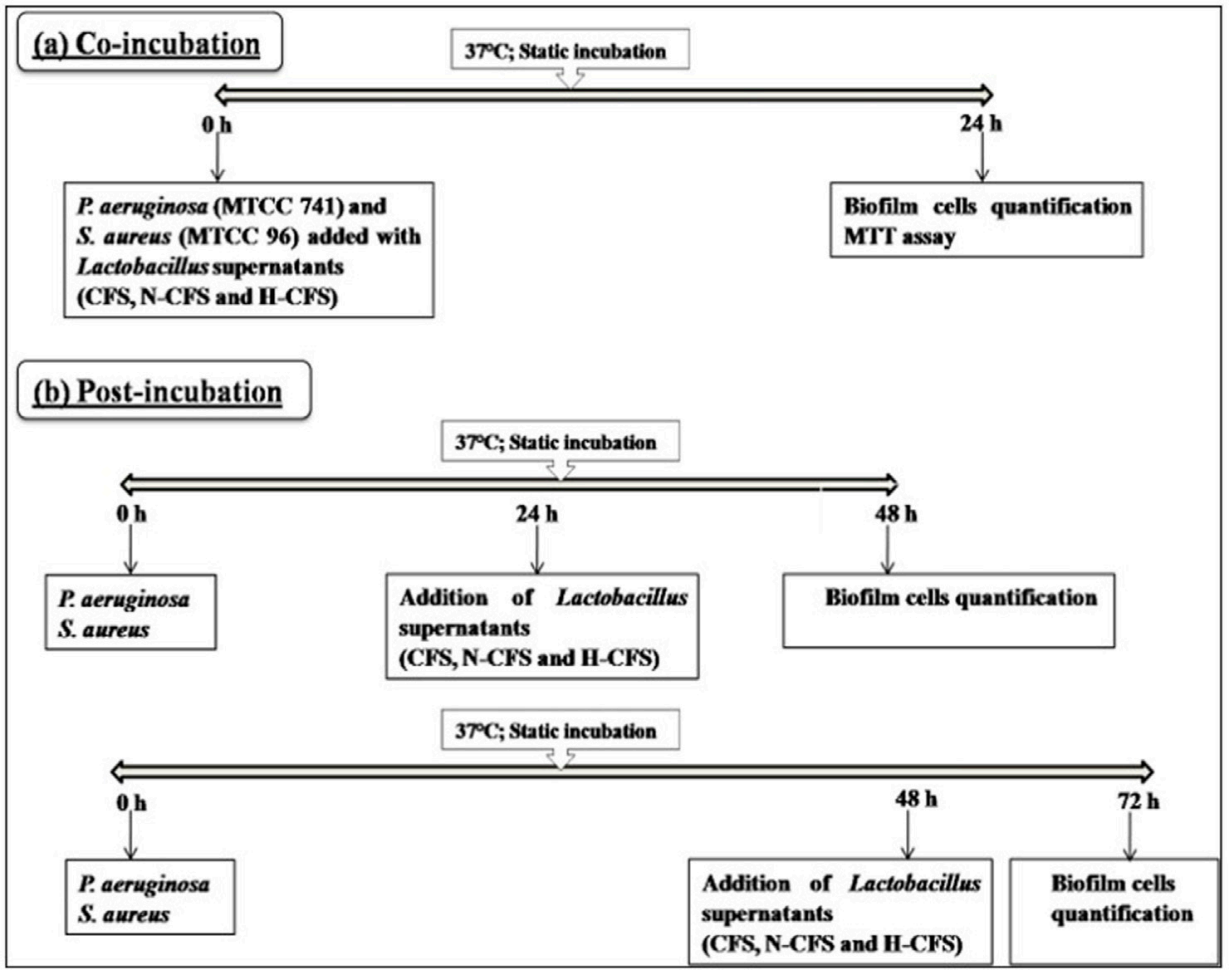
Flowchart of the biofilm inhibition assay via **(A)** co-incubation and **(B)** post-incubation strategy.

The anti-biofilm potential of LAB cell-free preparations was determined in pre-sterilized, polystyrene, flat-bottom 96-well microtiter plates (MTPs; Thermo Fischer, United States) using the crystal violet (CV; Fischer Scientific, India) staining assay (Zago et al., 2015). In brief, 10 μL of *S. aureus* (∼10^6^ CFU/mL) and *P. aeruginosa* (∼10^6^ CFU/mL) cell suspensions were incubated with CFS, N-CFS, and H-CFS preparations (30 μL each) using two different strategies, as illustrated in Figure 1 and described below.

### 2.4 Inhibition of cell attachment and biofilm formation (co-incubation)

To study the effect of CFS preparations on initial adhesion of test pathogens, the LAB cell-free preparations (∼10^9^ CFU/mL) and pathogen cells (10 μL, ∼10^6^ CFU/mL) were seeded simultaneously into MTPs and incubated for 24 h at 37°C.

### 2.5 Eradication of the pre-formed biofilm (post-incubation)

An aliquot of 10 μL of active cell suspensions of *S. aureus* (∼10^6^ CFU/mL) and *P. aeruginosa* (∼10^6^ CFU/mL) in BHI broth (30 μL) was seeded in MTPs and allowed to develop biofilm for 24 and 48 h at 37°C. Following incubation, developed biofilms were challenged with CFS preparations from LAB (Singh et al., 2018) for 24 h (Figure 1).

In both strategies, incubation and CFS challenge were followed by the aspiration of cell suspensions and washing of biofilms with phosphate-buffered saline (1X PBS) (200 μL, twice) to remove loosely adherent cells. The surface-adhered cells were then stained with 200 μL of the 0.2% CV solution for 15 min at room temperature. Following staining, the CV solution was decanted, and the stained cells were solubilized with 33% glacial acetic acid (250 μL) (Merck, India). The biofilm biomass was then quantified by measuring the intensity of CV at OD_570nm_ using a GO Skan microplate reader (Thermo Fisher Scientific, United States). To examine whether any of the constituents of pure MRS broth contribute to the anti-biofilm activity, MRS broth and its heat-treated preparation (H-MRS) were also tested along with CFS preparations. The CV dye used in this study is non-selective and binds negatively charged molecules of bacteria and EPS, without differentiating between live and dead cells. Hence, the observations made cannot be directly extrapolated to the eradication of the preformed biofilm; however, they can serve as indicative findings. The anti-biofilm activity (%) of different LAB cell-free preparations was calculated as follows: anti-biofilm activity (%) = (Control _OD570 nm_ − Test _OD570 nm_/Control _OD570 nm_) × 100. The positive control was the amount of biofilm formed with a pure culture of *S. aureus* and *P. aeruginosa*, whereas sterile BHI served as the negative control.

### 2.6 Determination of antibacterial activity of cell-free supernatants and impact on cell viability in bacterial biofilms

The antibacterial activity of cell-free preparations was determined using the agar-well diffusion assay (Sharma et al., 2017). In brief, 100 μL of an overnight-grown pathogen bacterial culture (10^6^ CFU/mL) was spread-plated onto BHI agar plates and allowed to air dry. Subsequently, 100 μL of CFSs was added to pre-marked wells, and the plates were incubated at 37°C for 24 h. The inhibition continuums were recorded.

The cell viability within biofilms was assessed using the formazan dye-based MTT (3-[4,5-dimethylthiazole-2-yl]-2,5-diphenyltetrazolium bromide; Sigma, India) assay in sterile 96-well polystyrene plates (Gomez-Flores et al., 1995). In brief, pathogens were allowed to grow in the presence or absence of CFS preparations in MTP for 24 h, as described earlier. Following incubation, 10 μL of the MTT dye was added to each well, and mixtures were further incubated for 4 h at 37°C*,* followed by the addition of 100 µL of dimethyl sulfoxide (DMSO) to the reaction mixture. Plates were further incubated for 30 min to permit the solubilization of crystallized formazan and were read at 570 *nm.* Percentage viability of cells was calculated using the following equation: percent viability = (absorbance of treated/absorbance of control) x 100.

### 2.7 Visualization of biofilms

#### 2.7.1 Light microscopic analysis

*S. aureus* and *P. aeruginosa* biofilms were allowed to develop on pre-sterilized glass (1 × 1 cm) cover slips. An aliquot of 80 μL/well (∼10^6^ CFU/mL) of overnight-grown test pathogens was added to 6-well tissue culture plates containing sterile glass cover slips, BHI medium (1,680 μL/well), and CFSs (240 μL/well). Wells without CFS served as control. Following 48 h of incubation at 37°C, planktonic cells were removed, and cover slips were rinsed with phosphate buffer (pH 6.5) and stained with the 0.2% CV solution for 30 min (Musthafa et al., 2010). The stained cover slips were visualized under a light microscope at ×40 magnification (Olympus Microscope, United States).

#### 2.7.2 Fluorescent microscopy

Pathogen biofilms, under different treatment conditions, were allowed to develop on pre-sterilized glass cover slips (1 × 1 cm), as described earlier. Biofilms were stained with the LIVE/DEAD BacLight Bacterial Viability Kit (L10316, Invitrogen-Molecular Probes, United States) as per the manufacturer’s instructions. The kit includes a mixture of two fluorescence dyes, namely, SYTO 9 (green-fluorescent nucleic acid stain) and propidium iodide (red-fluorescent nucleic acid stain). Bacterial cells with healthy or intact membranes stain green, whereas dead cells or cells with damaged membranes stain fluorescent red. The stained biofilms were observed at ×400 magnification using a fluorescence microscope (Olympus Microscopy, United States) equipped with an imaging system QIClick™ (Olympus, United States).

### 2.8 Statistical analysis

Each trial was conducted in triplicate on three different occasions with independently grown cultures unless otherwise stated, and data were represented as mean values (n = 3). Statistical significance was established by ANOVA and Tukey’s multiple comparison test (*p* < 0.05) using SPSS version 21.0 (SPSS Inc., Chicago, IL, United States). To check for the efficacy of LAB CFSs to inhibit pathogen biofilm formation under co- and post-incubation strategies, principal component analysis (PCA) was applied to the dataset through multivariate exploratory techniques using XLSTAT software version 2017.03 (ADDINSOFT SARL, Paris, France). The most promising isolates from breast milk and infant fecal were further compared with the reference probiotics *L. rhamnosus* GG and *L. casei* using PCA.

## 3 Results

### 3.1 Anti-biofilm potential of cell-free supernatants from LAB

Cell-free preparations from LAB strains from breast milk and infant fecal origin were assessed for their anti-biofilm activity against *S. aureus* and *P. aeruginosa*. The data pertaining to the reduction in biofilm biomass assayed by CV staining are presented in Tables 2, 3. The results indicated that the anti-biofilm activity significantly varied with the LAB strains, type of cell-free preparations (CFS, N-CFS, and H-CFS), and the co-incubation and post-incubation treatment strategies adopted. A negative control (fresh MRS and BHI medium incubated with tested pathogens) was used for each test. No inhibition was observed in any case of the untreated control.

**TABLE 2:** Effects of un-treated CFS, neutralized CFS (N-CFS) and heat-treated CFS (H-CFS) on the biofilm inhibition of *Staphylococcus aureus*. Values are expressed in percentage of biofilm inhibition in relation to the control.

Isolates		Co-incubation	Post-incubation		Isolates		Co-incubation	Post-incubation	
		0 h old biofilm	24 h old biofilm	48 h old biofilm			0 h old biofilm	24 h old biofilm	48 h old biofilm
HM1	CFS	74.27^f^ ± 3.71	54.23^m^ ± 2.21	NE^a^	IF1	CFS	73.78^k^ ± 3.58	17.16^f,g^ ± 1.72	69.11^o^ ± 2.87
	N-CFS	65.75^d^ ± 3.11	22.45^g,h^ ± 1.98	NE^a^		N-CFS	NE^a^	NE^a^	NE^a^
	H-CFS	69.78^e^ ± 2.82	37.24^j^ ± 1.45	NE^a^		H-CFS	79.72^l^ ± 3.62	10.37^d,e^ ± 1.21	50.87^k,l^ ± 2.13
HM2	CFS	NE^a^	25.50^h,i^ ± 1.91	24.62^c^ ± 1.84	IF2	CFS	52.78^j^ ± 2.84	48.91^k^ ± 2.51	65.63^n,o^ ± 3.81
	N-CFS	NE^a^	NE^a^	NE^a^		N-CFS	NE^a^	NE^a^	0.84^a^ ± 0.09
	H-CFS	NE^a^	14.64^d,e,f^ ± 1.07	11.86^b^ ± 1.02		H-CFS	51.87^j^ ± 3.13	18.47^f,g,h^ ± 1.42	53.15^m^ ± 1.63
HM3	CFS	NE^a^	16.50^d,e,f^ ± 1.08	67.12^m^ ± 2.84	IF3	CFS	NE^a^	3.80^a,b^ ± 0.04	37.11^i,j^ ± 1.65
	N-CFS	NE^a^	NE^a^	3.78^a^ ± 0.81		N-CFS	NE^a^	NE^a^	11.87^b,c,d^ ± 1.21
	H-CFS	NE^a^	15.62^d,e,f^ ± 1.13	32.20^d^ ± 1.78		H-CFS	NE^a^	2.02^a,b^ ± 0.11	13.99^c,d,e^ ± 1.67
HM6	CFS	NE^a^	25.32^h,i^ ± 1.70	60.37^k,l^ ± 2.84	IF4	CFS	NE^a^	NE^a^	45.80^k^ ± 2.31
	N-CFS	NE^a^	NE^a^	NE^a^		N-CFS	NE^a^	NE^a^	32.95^i^ ± 2.11
	H-CFS	NE^a^	16.95^e,f^ ± 1.43	37.33^e,f^ ± 2.11		H-CFS	18.16^f,g^ ± 1.67	5.73^b,c,a^ ± 0.24	39.50^j^ ± 2.96
HM7	CFS	NE^a^	11.92^c,d^ ± 1.73	62.27^k,l,m^ ± 3.71	IF5	CFS	NE^a^	NE^a^	4.71^a,b^ ± 0.08
	N-CFS	NE^a^	NE^a^	NE^a^		N-CFS	NE^a^	NE^a^	0.42^a^ ± 0.02
	H-CFS	NE^a^	6.81^b^ ± 1.09	33.34^d,e^ ± 1.91		H-CFS	NE^a^	7.37^c,d^ ± 0.83	3.67^a,b^ ± 0.01
HM8	CFS	48.29^c^ ± 1.53	28.52^i^ ± 2.16	49.70^i,j^ ± 2.31	IF6	CFS	NE^a^	6.29^b,c^ ± 0.73	22.65^g,h^ ± 1.46
	N-CFS	NE^a^	NE^a^	1.59^a^ ± 0.03		N-CFS	NE^a^	NE^a^	NE^a^
	H-CFS	NE^a^	NE^a^	39.93^f,g^ ± 1.21		H-CFS	NE^a^	NE^a^	24.34^h^ ± 1.54
HM9	CFS	5.98^b^ ± 0.41	10.00^b,c^ ± 1.17	59.36^k^ ± 2.91	IF7	CFS	16.80^e,f^ ± 1.52	1.24^a,b^ ± 0.56	26.02^h^ ± 1.05
	N-CFS	NE^a^	NE^a^	NE^a^		N-CFS	NE^a^	NE^a^	0.37^a^ ± 0.07
	H-CFS	NE^a^	NE^a^	15.91^b^ ± 1.61		H-CFS	10.22^c^ ± 1.46	NE^a^	25.59^h^ ± 1.42
HM10	CFS	NE^a^	50.67^l,m^ ± 2.84	64.38^l,m^ ± 2.72	IF8	CFS	NE^a^	54.41^l^ ± 2.86	34.78^i^ ± 2.65
	N-CFS	NE^a^	NE^a^	NE^a^		N-CFS	14.47^d,e^ ± 1.93	NE^a^	NE^a^
	H-CFS	NE^a^	23.59^g,h^ ± 1.52	45.11^h,i^ ± 3.11		H-CFS	12.42^c,d^ ± 1.84	15.80^f^ ± 1.81	19.35^f,g^ ± 1.63
HM11	CFS	NE^a^	22.71^g,h^ ± 1.92	52.32^j^ ± 2.77	IF9	CFS	79.08^l^ ± 3.42	27.57^i^ ± 1.63	66.46^n,o^ ± 3.46
	N-CFS	NE^a^	NE^a^	NE^a^		N-CFS	52.34^j^ ± 2.74	NE^a^	NE^a^
	H-CFS	NE^a^	19.98^e,f^ ± 1.52	35.40^d,e,f^ ± 1.73		H-CFS	85.19^m^ ± 3.65	49.14^k^ ± 2.81	50.42^l,m^ ± 2.31
HM12	CFS	NE^a^	48.73^k,l^ ± 2.75	46.35^h,i^ ± 1.29	IF10	CFS	NE^a^	NE^a^	24.73^h^ ± 1.85
	N-CFS	NE^a^	NE^a^	NE^a^		N-CFS	NE^a^	NE^a^	17.84^e,f^ ± 1.93
	H-CFS	NE^a^	35.24^j^ ± 2.01	44.09^g,h^ ± 2.82		H-CFS	20.84^g^ ± 1.23	11.57^e^ ± 1.82	15.37^d,e,f^ ± 1.32
HM13	CFS	50.63^c^ ± 1.42	44.19^k^ ± 2.81	66.39^m^ ± 3.91	IF11	CFS	73.40^k^ ± 3.81	55.42^l^ ± 2.92	47.85^k,l^ ± 2.71
	N-CFS	NE^a^	NE^a^	0.87^a^ ± 0.04		N-CFS	NE^a^	NE^a^	NE^a^
	H-CFS	NE^a^	38.11^j^ ± 1.99	61.18^i,m,n^ ± 3.63		H-CFS	78.54^l^ ± 3.28	57.38^l^ ± 3.09	41.14^j^ ± 2.89
					IF12	CFS	NE^a^	55.72^l^ ± 2.83	63.53^n^ ± 3.63
						N-CFS	NE^a^	NE^a^	15.29^d,e,f^ ± 1.56
						H-CFS	NE^a^	48.91^k^ ± 2.07	10.59 ± ^b,c1.11^
					IF13	CFS	47.99^i^ ± 2.71	44.48^j^ ± 2.58	12.17^b,c,d^ ± 1.09
						N-CFS	NE^a^	NE^a^	NE^a^
						H-CFS	NE^a^	30.01^g,h^ ± 1.56	14.33^c,d,e^ ± 1.14
					IF14	CFS	25.21^i^ ± 1.41	50.18^k^ ± 2.53	37.94^j^ ± 2.51
						N-CFS	NE^a^	NE^a^	NE^a^
						H-CFS	10.74^c^ ± 1.06	9.85^d,e^ ± 1.03	9.12^b^ ± 1.32
					IF15	CFS	NE^a^	21.13^h^ ± 1.92	41.47^j^ ± 2.84
						N-CFS	NE^a^	15.82^h^ ± 1.04	NE^a^
						H-CFS	3.80^b^ ± 0.02	9.09^c,d,e^ ± 0.93	20.29^f,g^ ± 1.52

NE implies No Effect; Alphabets a–o means in the column with same superscript letter are not significantly different as measured by 2 sided Tukey’s – post-hoc range test between replications.

**TABLE 3:** Effects of un-treated CFS, neutralized CFS (N-CFS) and heat-treated CFS (H-CFS) on the biofilm inhibition of *Pseudomonas aeruginosa*. Values are expressed in percentage of biofilm inhibition in relation to the control.

Isolates		Co-incubation	Post-incubation		Isolates		Co-incubation	Post-incubation	
		0 h old biofilm	24 h old biofilm	48 h old biofilm			0 h old biofilm	24 h old biofilm	48 h old biofilm
HM1	CFS	42.06^m,n^ ± 1.47	59.56^h^ ± 3.05	NE^a^	IF1	CFS	29.39^c,d,e,f,g^ ± 2.54	NE^a^	NE^a^
	N-CFS	38.81^k,l,m^ ± 1.84	54.25^g^ ± 2.78	NE^a^		N-CFS	25.01^e,f,g,h,i,j^ ± 1.96	14.74^i^ ± 1.91	NE^a^
	H-CFS	32.28^h,i,j,k^ ± 1.56	50.88^g^ ± 1.94	NE^a^		H-CFS	19.33^c,d,e,f,g,h^ ± 1.64	2.74^a,b,c^ ± 0.92	NE^a^
HM2	CFS	42.90^n^ ± 2.57	NE^a^	1.31^b^ ± 0.83	IF2	CFS	30.57h^i,j,k,l^ ± 2.31	NE^a^	NE^a^
	N-CFS	6.73^b^ ± 0.04	NE^a^	NE^a^		N-CFS	11.54^a,b,c,d^ ± 1.10	14.90^i^ ± 1.42	NE^a^
	H-CFS	33.49^g,h,i,j^ ± 1.11	NE^a^	NE^a^		H-CFS	13.31^b,c,d,e^ ± 1.14	NE^a^	NE^a^
HM3	CFS	25.90^e,f^ ± 1.86	NE^a^	NE^a^	IF3	CFS	27.05^g,h,i,j,k^ ± 1.84	7.51^e,f,g^ ± 1.13	NE^a^
	N-CFS	NE^a^	NE^a^	NE^a^		N-CFS	NE^a^	NE^a^	NE^a^
	H-CFS	7.41^b^ ± 0.52	NE^a^	NE^a^		H-CFS	NE^a^	NE^a^	NE^a^
HM6	CFS	33.32^h,i,j,k^ ± 1.25	9.46^c,d^ ± 0.18	NE^a^	IF4	CFS	NE^a^	8.55^e,f,g^ ± 1.61	NE^a^
	N-CFS	NE^a^	NE^a^	NE^a^		N-CFS	7.77^a,b,c^ ± 0.24	7.31^d,e,f,g^ ± 0.95	NE^a^
	H-CFS	20.99^c,d,e^ ± 2.15	NE^a^	NE^a^		H-CFS	NE^a^	NE^a^	NE^a^
HM7	CFS	20.89^c,d,e^ ± 1.95	7.59^c,d^ ± 0.56	NE^a^	IF5	CFS	37.39^k,l^ ± 1.69	2.67^a,b,c^ ± 0.17	NE^a^
	N-CFS	NE^a^	NE^a^	NE^a^		N-CFS	NE^a^	NE^a^	NE^a^
	H-CFS	16.85^c^ ± 1.35	NE^a^	NE^a^		H-CFS	15.82^c,d,e,f,g^ ± 1.58	NE^a^	NE^a^
HM8	CFS	34.45^i,j,k,l^ ± 1.95	5.80^b,c,d^ ± 0.24	NE^a^	IF6	CFS	29.70^h,i,j,k,l^ ± 1.64	NE^a^	NE^a^
	N-CFS	NE^a^	NE^a^	NE^a^		N-CFS	NE^a^	NE^a^	NE^a^
	H-CFS	NE^a^	NE^a^	NE^a^		H-CFS	NE^a^	NE^a^	NE^a^
HM9	CFS	26.96^f,g,h^ ± 1.82	1.01^a,b^ ± 0.09	NE^a^	IF7	CFS	NE^a^	NE^a^	NE^a^
	N-CFS	NE^a^	NE^a^	NE^a^		N-CFS	NE^a^	NE^a^	NE^a^
	H-CFS	NE^a^	NE^a^	NE^a^		H-CFS	NE^a^	NE^a^	NE^a^
HM10	CFS	NE^a^	1.23^a,b^ ± 0.03	NE^a^	IF8	CFS	35.27^j,k,l^ ± 2.51	NE^a^	NE^a^
	N-CFS	20.01^c,d^ ± 1.23	9.25^c,d^ ± 0.07	NE^a^		N-CFS	NE^a^	NE^a^	NE^a^
	H-CFS	25.08^d,e,f^ ± 1.54	NE^a^	NE^a^		H-CFS	16.51^c,d,e,f,g^ ± 1.17	4.13^b,c,d^ ± 0.03	NE^a^
HM11	CFS	33.57^h,i,j,k^ ± 2.68	NE^a^	NE^a^	IF9	CFS	34.07^i,j,k,l^ ± 1.69	16.89^i,j^ ± 1.04	NE^a^
	N-CFS	24.75^d,e,f^ ± 2.34	NE^a^	NE^a^		N-CFS	14.63^c,d,e,f^ ± 1.05	NE^a^	NE^a^
	H-CFS	28.50^g,h,i^ ± 1.34	8.88^c,d^ ± 0.94	NE^a^		H-CFS	15.04^c,d,e,f,g^ ± 1.54	NE^a^	NE^a^
HM12	CFS	36.89^j,k,l,m^ ± 2.31	1.15^a,b^ ± 0.21	NE^a^	IF10	CFS	26.52^f,g,h,i,j,k^ ± 1.38	22.66^j^ ± 1.11	NE^a^
	N-CFS	4.75^a,b^ ± 0.09	NE^a^	2.04^b^ ± 0.40		N-CFS	NE^a^	9.91^g,h^ ± 1.02	NE^a^
	H-CFS	18.82^c^ ± 1.10	NE^a^	NE^a^		H-CFS	15.29^c,d,e,f,g^ ± 1.37	9.15^f,g^ ± 0.93	NE^a^
HM13	CFS	31.51^ghi^ ± 1.05	NE^a^	NE^a^	IF11	CFS	24.06^e,f,g,h,i,j^ ± 1.52	19.71^j,k^ ± 0.85	NE^a^
	N-CFS	NE^a^	14.85^e,f^ ± 1.09	NE^a^		N-CFS	NE^a^	NE^a^	NE^a^
	H-CFS	39.38^c,m,n^ ± 2.18	NE^a^	NE^a^		H-CFS	16.13^c,d,e,f,g^ ± 1.05	NE^a^	NE^a^
					IF12	CFS	33.07^i,j,k,l^ ± 1.51	NE^a^	NE^a^
						N-CFS	1.66^a,b^ ± 0.05	NE^a^	NE^a^
						H-CFS	22.15^d,e,f,g,h,i^ ± 1.72	NE^a^	NE^a^
					IF13	CFS	51.51^m,n^ ± 2.84	11.85^h,i^ ± 1.24	1.05^b^ ± 0.11
						N-CFS	41.55l^m^ ± 2.91	3.99^b,c,d^ ± 0.04	NE^a^
						H-CFS	16.96^c,d,e,f,g^ ± 1.17	NE^a^	NE^a^
					IF14	CFS	41.80^l,m^ ± 2.41	NE^a^	NE^a^
						N-CFS	35.82^j,k,^ ± 1.98	NE^a^	0.74^a,b^ ± 0.01
						H-CFS	49.90^l,m^ ± 2.65	NE^a^	NE^a^
					IF15	CFS	56.56^m,n^ ± 2.34	NE^a^	NE^a^
						N-CFS	8.75^a,b,c^ ± 0.74	NE^a^	NE^a^
						H-CFS	NE^a^	NE^a^	NE^a^

NE implies No Effect; Alphabets a–n means in the column with same superscript letter are not significantly different as measured by 2 sided Tukey’s – post-hoc range test between replications.

### 3.2 Inhibition of cell attachment and biofilm formation

The biofilm inactivation pattern varied with the untreated, neutralized, and heat-treated CFS preparations (Tables 2, 3). The LAB supernatants reduced pathogen cell attachment to the polystyrene surface. After a 24-hour challenge, only HM1, IF9, and IF11 were able to significantly (p < 0.05) retard the production of *S. aureus* biofilms (52.34%–85.19%) using all three cell-free preparations (Table 2). Furthermore, it is interesting that HM1, IF9, and IF8 show anti-biofilm action among the N-CFS preparations. Conversely, all three of the cell-free preparations of a total of 10 LAB strains (HM1, HM2, HM11, HM12, IF1, IF2, IF9, IF12, IF13, and IF14) reduced *P. aeruginosa* biofilm formation, albeit to a moderate extent (25.01%–51.51%) (Table 3). A varied range of biofilm impairment was reported with different cell-free preparations of other isolates against both *S. aureus* and *P. aeruginosa*. Although N-CFS and H-CFS preparations of few LAB strains significantly (p < 0.05) inhibited the biofilm formation by test pathogens, the percent reduction was not as great as that observed with their untreated forms. To check whether uninoculated fresh MRS or its heat-treated preparation possesses any anti-biofilm activity, fresh MRS and H-MRS were incubated with test pathogens, and no significant inhibition was recorded.

### 3.3 Eradication of the preformed biofilm

The anti-biofilm effects of CFSs were further investigated against mature biofilms. *S. aureus* and *P. aeruginosa* were allowed to develop biofilm for different time intervals (24 and 48 h), followed by a challenge with bacterial CFS preparations for 24 h (Figure 1). Interestingly, CFSs could also impair pre-formed biofilm structures to varied degrees. Only HM1 (54.23%) of the three cell-free preparations was able to clearly suppress the *S. aureus* biofilm that had been growing for 24 h (Table 2). In contrast, LAB CFSs, whether treated or untreated, displayed no significant anti-biofilm effect against the pre-formed (24 and 48 h old) biofilm of *P. aeruginosa* (Table 3). Among the tested LAB isolates, only HM1 (50.88%–59.56%) and IF10 (9.15%–22.66%) could impair the 24-h-old biofilm of *P. aeruginosa*, with all the three cell-free preparations. In contrast to *S. aureus*, most strains failed to inactivate the 48-h-old biofilm of *P. aeruginosa*.

A comparative analysis of the results from both sets of experiments revealed that breast milk and infant fecal origin isolates, *viz*., HM1, HM8, HM13, IF1, IF9, and IF11 against *S. aureus* and HM1, HM2, HM11, IF13, IF14, and IF15 against *P. aeruginosa*, presented the most promising anti-biofilm activity. These strains were shortlisted and further statistically compared to the reference probiotic strains, *viz*., *L. rhamnosus* GG and *L. casei*, for a more rational overview (Table 4).

### 3.4 Antibacterial activity of cell-free supernatants and impact on cell viability in bacterial biofilms

The antibacterial effects of CFS of LAB isolates were determined by measuring the zone of growth inhibition of *S. aureus* and *P. aeruginosa*. As documented in the previous study by our research group, breast milk isolates display moderate-to-weak antibacterial activity against *P. aeruginosa*, while no antibacterial effects were reported against *S. aureus* (Sharma et al., 2017). In the present study, isolates of infant fecal origin were screened for their antagonistic activity against the test pathogens. Interestingly, the antagonistic activity of overnight CFSs increased with extended incubation of 24 and 48 h, displaying moderate inhibition. However, the antibacterial effects disappeared, following pH neutralization and heat treatment. The results of *in vitro* antibacterial activity using the well diffusion assay strongly correlate with the impact on cell viability observed in the MTT assay.

To further study the impact of CFSs on *S. aureus* and *P. aeruginosa* viability, an MTT assay was performed, following a 24 h exposure of old biofilms to treated and untreated cell-free preparations (Table 5). The MTT assay measures the enzymatic activity of actively respiring cells and, thus, denotes relative numbers of viable cells in bacterial biofilms. Fluorescent microscopic images, following the challenge of pathogen biofilms with CFSs for 24 h, indicated reduced cell viability. Among breast milk isolates, the viability of *S. aureus* cells was most promisingly reduced by HM1, as recorded with the percent viability of 50.31, 68.27, and 46.28, with untreated CFS, N-CFS, and H-CFS preparations, respectively. The viability of *P. aeruginosa*, upon the challenge with breast milk LAB cell-free preparations, ranged from 41.61% (HM1 N-CFS) to 94.59% (HM3 N-CFS). The results indicated that the *S. aureus* cells were more sensitive to the breast milk LAB CFS challenge than *P. aeruginosa.* In contrast, the CFSs of infant fecal origin lactobacilli did not display much antagonistic activity against both the pathogens, as recorded with the high cell viability values varying from 50.38% to 98.37% for *S. aureus* and 47.01% to 98.90% for *P. aeruginosa*.

**TABLE 4:** Biofilm inhibitory comparative studies of best promising strains with reference strains against *Pseudomonas aeruginosa* (A) and *Staphylococcus aureus* (B).

(A) Isolates		Co-incubation	Post-incubation	
		0 h old biofilm	24 h old biofilm	48 h old biofilm
*L. rhamnosus* GG	CFS	79.92^m^ ± 2.67	57.53^i,j^ ± 1.57	NE^a^
	N-CFS	60.41^k^ ± 1.99	50.79^h^ ± 1.96	3.79^b^ ± 0.24
	H-CFS	51.15^i,j^ ± 2.34	NE^a^	NE^a^
*L. casei*	CFS	71.82^l^ ± 3.24	43.65^g^ ± 1.58	NE^a^
	N-CFS	59.82^k^ ± 2.45	39.68^f^ ± 1.25	46.34^c^ ± 1.64
	H-CFS	34.57^f^ ± 1.29	NE^a^	NE^a^
HM1	CFS	42.06^g,h^ ± 1.47	59.56^j^ ± 3.05	NE^a^
	N-CFS	38.81^f,g^ ± 1.84	54.25^h,i^ ± 2.78	NE^a^
	H-CFS	32.28^e,g^ ± 1.56	50.88^h^ ± 1.94	NE^a^
HM2	CFS	42.9^h^ ± 2.57	NE^a^	1.31^a,b^ ± 0.83
	N-CFS	6.73^b^ ± 0.04	NE^a^	NE^a^
	H-CFS	33.49^e,f^ ± 1.11	NE^a^	NE^a^
HM11	CFS	33.57^e,f^ ± 2.68	NE^a^	NE^a^
	N-CFS	24.75^d^ ± 2.34	NE^a^	NE^a^
	H-CFS	28.51^d,e^ ± 1.34	8.88^c^ ± 0.94	NE^a^
IF13	CFS	51.51^i,j^ ± 2.84	11.85^d,e^ ± 1.24	1.05^a,b^ ± 0.11
	N-CFS	41.55^g,h^ ± 2.91	3.99^b^ ± 0.04	NE^a^
	H-CFS	16.96^c^ ± 1.17	NE^a^	NE^a^
IF14	CFS	41.80^g,h^ ± 2.41	NE^a^	NE^a^
	N-CFS	35.82^f^ ± 1.98	NE^a^	NE^a^
	H-CFS	49.90^i^ ± 2.65	NE^a^	0.74^a^ ± 0.01
IF15	CFS	56.56^j,k^ ± 2.34	NE^a^	NE^a^
	N-CFS	8.75^b^ ± 0.74	NE^a^	NE^a^
	H-CFS	NE^a^	NE^a^	NE^a^

(B) Isolates		Co-incubation	Post-incubation	
		0 h old biofilm	24 h old biofilm	48 h old biofilm
*L. rhamnosus* GG	CFS	71.00^g^ ± 0.91	35.62^g^ ± 0.47	NE^a^
	N-CFS	36.41^b^ ± 1.31	36.13^g^ ± 0.94	NE^a^
	H-CFS	56.23^d^ ± 0.77	14.24^c,d^ ± 0.27	NE^a^
*L. casei*	CFS	71.00^g^ ± 1.87	10.68^b,c^ ± 0.16	NE^a^
	N-CFS	60.81^e^ ± 0.84	9.92^b,c^ ± 0.04	NE^a^
	H-CFS	40.96^b^ ± 0.97	8.04^b^ ± 0.01	NE^a^
HM1	CFS	74.27^g,h,i^ ± 3.71	54.23^i,j^ ± 2.21	NE^a^
	N-CFS	65.75^f^ ± 3.11	22.45^e^ ± 1.98	NE^a^
	H-CFS	69.78^f,g^ ± 2.82	37.24^g^ ± 1.45	NE^a^
HM8	CFS	48.29^c^ ± 1.53	28.52^f^ ± 2.16	49.7^c^ ± 2.31
	N-CFS	NE^a^	NE^a^	1.59^a^ ± 0.03
	H-CFS	NE^a^	NE^a^	39.93^b^ ± 1.21
HM13	CFS	50.63^c,d^ ± 1.42	44.19^h^ ± 2.81	66.39^e^ ± 3.91
	N-CFS	NE^a^	NE^a^	0.87^a^ ± 0.04
	H-CFS	NE^a^	38.11^g^ ± 1.99	61.18^d^ ± 3.63
IF1	CFS	73.78^g,h^ ± 3.58	17.16^d^ ± 1.72	69.11^e^ ± 2.87
	N-CFS	NE^a^	NE^a^	NE^a^
	H-CFS	79.72^j^ ± 3.62	10.37^b,c^ ± 1.21	50.87^c^ ± 2.13
IF9	CFS	79.08^i,j^ ± 3.42	27.57^e,f^ ± 1.63	66.46^e^ ± 3.46
	N-CFS	52.34^c,d^ ± 2.74	NE^a^	NE^a^
	H-CFS	85.19^k^ ± 3.65	49.14^h,i^ ± 2.81	50.42^c^ ± 2.31
IF11	CFS	73.40^g^ ± 3.81	55.42^j^ ± 2.92	47.85^c^ ± 2.71
	N-CFS	NE^a^	NE^a^	NE^a^
	H-CFS	78.54^h,i,j^ ± 3.28	57.38^j^ ± 3.09	41.14^b^ ± 2.89

**TABLE 5:** Effect of the cell-free supernatants (CFSs) of isolates from breast milk (A) and infant faecal (B) *Lactobacillus* strains on the cell viability of *Staphylococcus aureus* and *Pseudomonas aeruginosa.*

(A) Breast milk isolates		Cell Viability (%)		Breast milk isolates		Cell Viability (%)		Breast milk isolates		Cell Viability (%)	
		*S. aureus*	*P. aeruginosa*			*S. aureus*	*P. aeruginosa*			*S. aureus*	*P. aeruginosa*
*L. rhamnosus* GG	CFS	29.54 ± 0.94	44.96 ± 1.71	HM6	CFS	88.25 ± 4.76	48.34 ± 2.34	HM10	CFS	70.56 ± 4.14	61.06 ± 3.16
	N-CFS	52.14 ± 1.41	52.34 ± 2.24		N-CFS	72.47 ± 3.69	89.80 ± 3.70		N-CFS	83.53 ± 3.73	89.84 ± 3.53
	H-CFS	48.37 ± 1.38	62.97 ± 2.81		H-CFS	70.88 ± 3.91	43.57 ± 2.73		H-CFS	67.38 ± 2.95	65.10 ± 2.95
*L. casei*	CFS	35.96 ± 1.29	53.12 ± 1.54	HM7	CFS	67.21 ± 2.76	49.91 ± 2.64	HM11	CFS	89.04 ± 3.48	49.05 ± 2.82
	N-CFS	52.17 ± 1.60	68.59 ± 2.28		N-CFS	84.51 ± 3.41	99.82 ± 4.76		N-CFS	73.57 ± 2.73	75.61 ± 3.15
	H-CFS	68.27 ± 2.91	71.06 ± 3.86		H-CFS	65.15 ± 2.94	50.13 ± 3.08		H-CFS	58.84 ± 2.18	60.70 ± 3.05
HM1	CFS	50.31 ± 2.31	76.84 ± 2.97	HM8	CFS	71.41 ± 3.94	58.19 ± 2.64	HM12	CFS	67.82 ± 2.39	50.59 ± 2.53
	N-CFS	68.27 ± 3.74	41.61 ± 2.13		N-CFS	85.22 ± 3.92	81.47 ± 3.03		N-CFS	90.72 ± 4.19	72.37 ± 2.86
	H-CFS	46.28 ± 2.83	78.24 ± 3.31		H-CFS	71.29 ± 3.56	62.88 ± 3.13		H-CFS	61.28 ± 3.21	47.69 ± 2.97
HM3	CFS	86.59 ± 3.97	43.07 ± 2.94	HM9	CFS	61.23 ± 2.96	48.84 ± 2.45	HM13	CFS	61.16 ± 2.02	47.91 ± 2.18
	N-CFS	96.52 ± 4.42	94.59 ± 4.95		N-CFS	67.23 ± 3.12	78.65 ± 3.37		N-CFS	81.40 ± 3.76	92.48 ± 4.75
	H-CFS	67.84 ± 3.68	47.58 ± 2.59		H-CFS	69.43 ± 3.06	48.69 ± 2.24		H-CFS	74.79 ± 3.84	52.21 ± 3.07

(B) Infant faecal isolates		Cell Viability (%)		Infant faecal isolates		Cell Viability (%)		Infant faecal isolates		Cell Viability (%)	
		*S. aureus*	*P. aeruginosa*			*S. aureus*	*P. aeruginosa*			*S. aureus*	*P. aeruginosa*
IF1	CFS	76.24 ± 1.41	58.60 ± 1.84	IF6	CFS	85.15 ± 3.68	85.46 ± 3.61	IF11	CFS	56.13 ± 2.36	48.64 ± 1.87
	N-CFS	80.25 ± 3.85	86.25 ± 2.76		N-CFS	85.25 ± 3.74	95.68 ± 4.85		N-CFS	84.62 ± 3.82	82.92 ± 3.65
	H-CFS	73.68 ± 2.97	61.00 ± 1.95		H-CFS	88.33 ± 2.97	76.90 ± 3.14		H-CFS	50.38 ± 2.33	75.46 ± 2.71
IF2	CFS	84.76 ± 2.31	47.01 ± 1.29	IF7	CFS	81.13 ± 3.38	91.96 ± 4.23	IF12	CFS	79.25 ± 3.47	68.22 ± 2.69
	N-CFS	91.72 ± 4.63	78.65 ± 2.78		N-CFS	94.10 ± 4.74	96.87 ± 4.14		N-CFS	82.97 ± 4.71	75.48 ± 3.14
	H-CFS	79.66 ± 3.78	70.26 ± 2.36		H-CFS	97.48 ± 4.96	95.34 ± 4.47		H-CFS	77.14 ± 3.96	72.89 ± 2.86
IF3	CFS	98.37 ± 4.96	74.28 ± 2.41	IF8	CFS	88.89 ± 3.78	76.64 ± 2.67	IF13	CFS	89.10 ± 4.81	59.94 ± 1.74
	N-CFS	78.86 ± 3.7	90.58 ± 3.82		N-CFS	89.92 ± 2.94	89.21 ± 3.64		N-CFS	93.70 ± 4.48	79.52 ± 2.12
	H-CFS	93.09 ± 4.27	64.58 ± 2.97		H-CFS	85.35 ± 3.16	70.85 ± 2.18		H-CFS	86.58 ± 3.69	67.30 ± 2.47
IF4	CFS	94.07 ± 4.93	78.01 ± 3.11	IF9	CFS	75.48 ± 2.54	81.55 ± 3.31	IF14	CFS	90.83 ± 4.11	76.73 ± 3.24
	N-CFS	80.12 ± 3.87	86.57 ± 3.82		N-CFS	92.39 ± 4.67	98.90 ± 4.16		N-CFS	96.35 ± 4.31	86.26 ± 3.82
	H-CFS	81.49 ± 2.37	86.52 ± 3.26		H-CFS	86.22 ± 3.64	55.32 ± 1.98		H-CFS	98.15 ± 4.85	78.27 ± 2.92
IF5	CFS	87.94 ± 3.47	91.04 ± 4.12	IF10	CFS	90.74 ± 4.18	81.04 ± 3.69	IF15	CFS	92.45 ± 3.96	86.38 ± 3.67
	N-CFS	90.25 ± 4.38	96.98 ± 4.82		N-CFS	89.41 ± 3.49	92.16 ± 4.17		N-CFS	98.06 ± 4.21	97.48 ± 4.86
	H-CFS	77.56 ± 2.71	90.04 ± 3.97		H-CFS	91.79 ± 4.25	85.10 ± 3.41		H-CFS	90.56 ± 3.94	98.81 ± 4.41

### 3.5 Microscopic images

**FIGURE 2:**
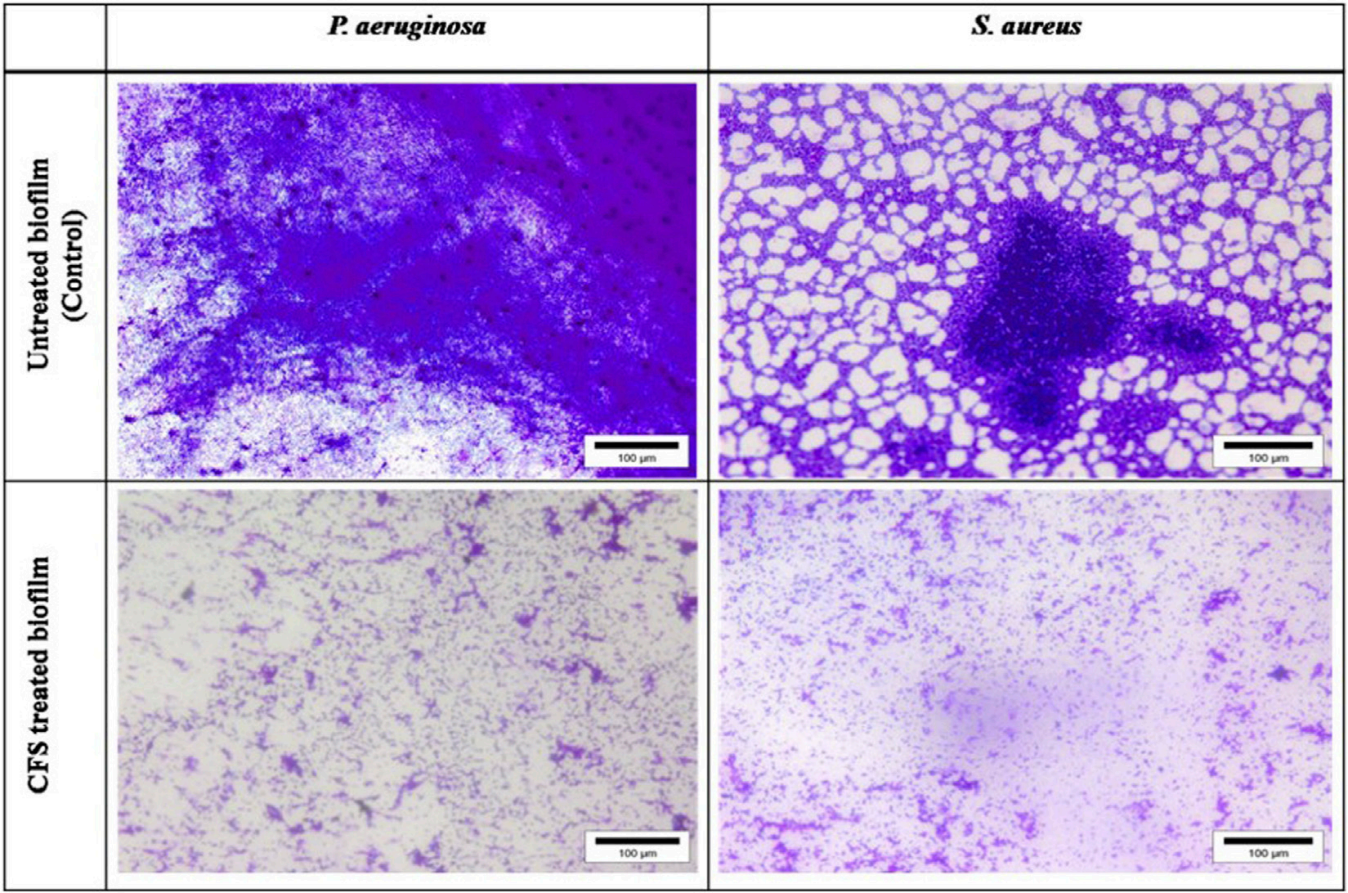
Light microscopic images of biofilms of *S. aureus* and *P. aeruginosa* grown in the absence and presence of un-treated cell free supernatant of lactobacilli.

**FIGURE 3:**
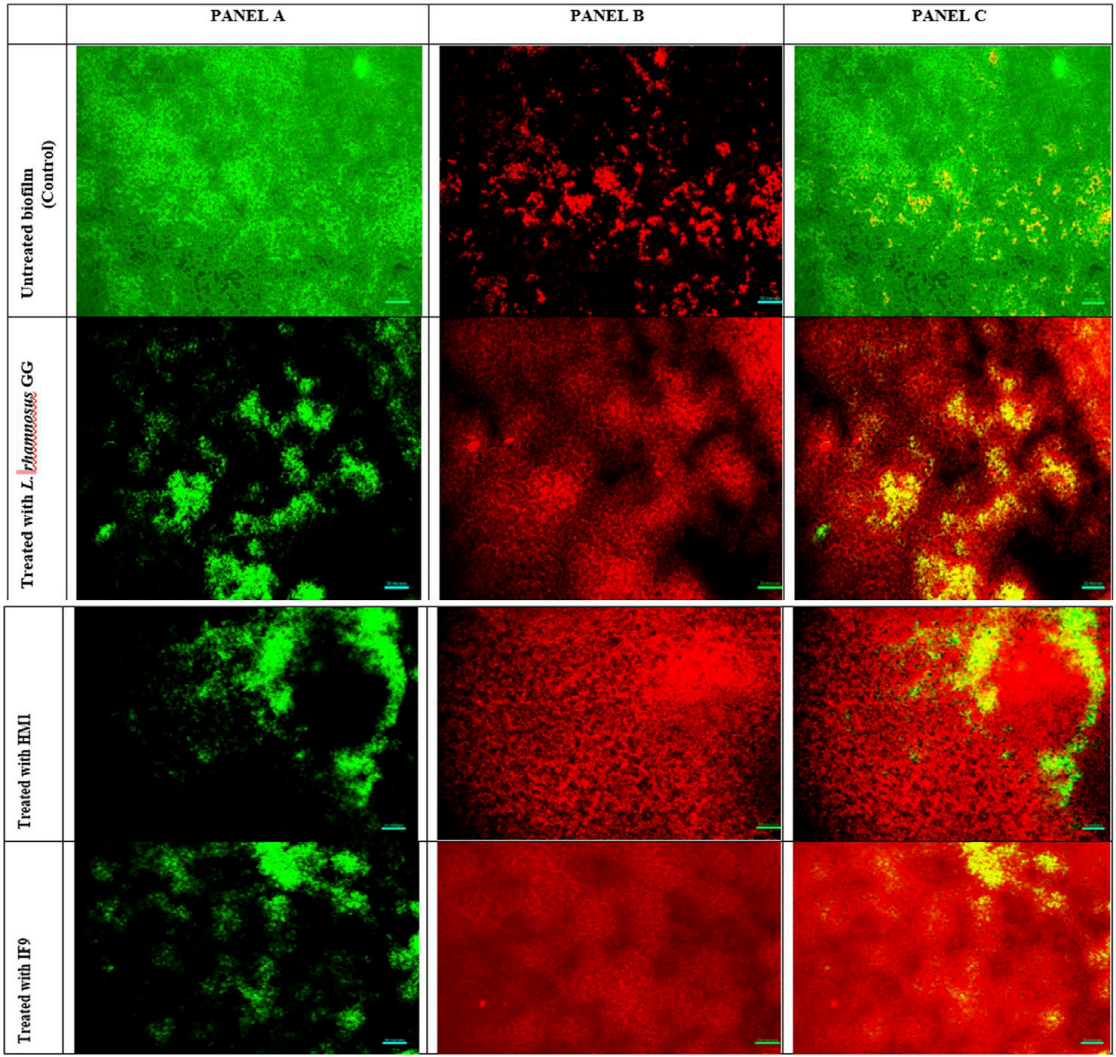
Fluorescent microscopic images of biofilms of *S. aureus* grown in the absence and presence of un-treated cell free supernatant of lactobacilli. Panel A (left) is the image obtained from the green channel (live cells), panel B (center) from the red channel (dead cells) and panel C (right) is a merged image.

**FIGURE 4:**
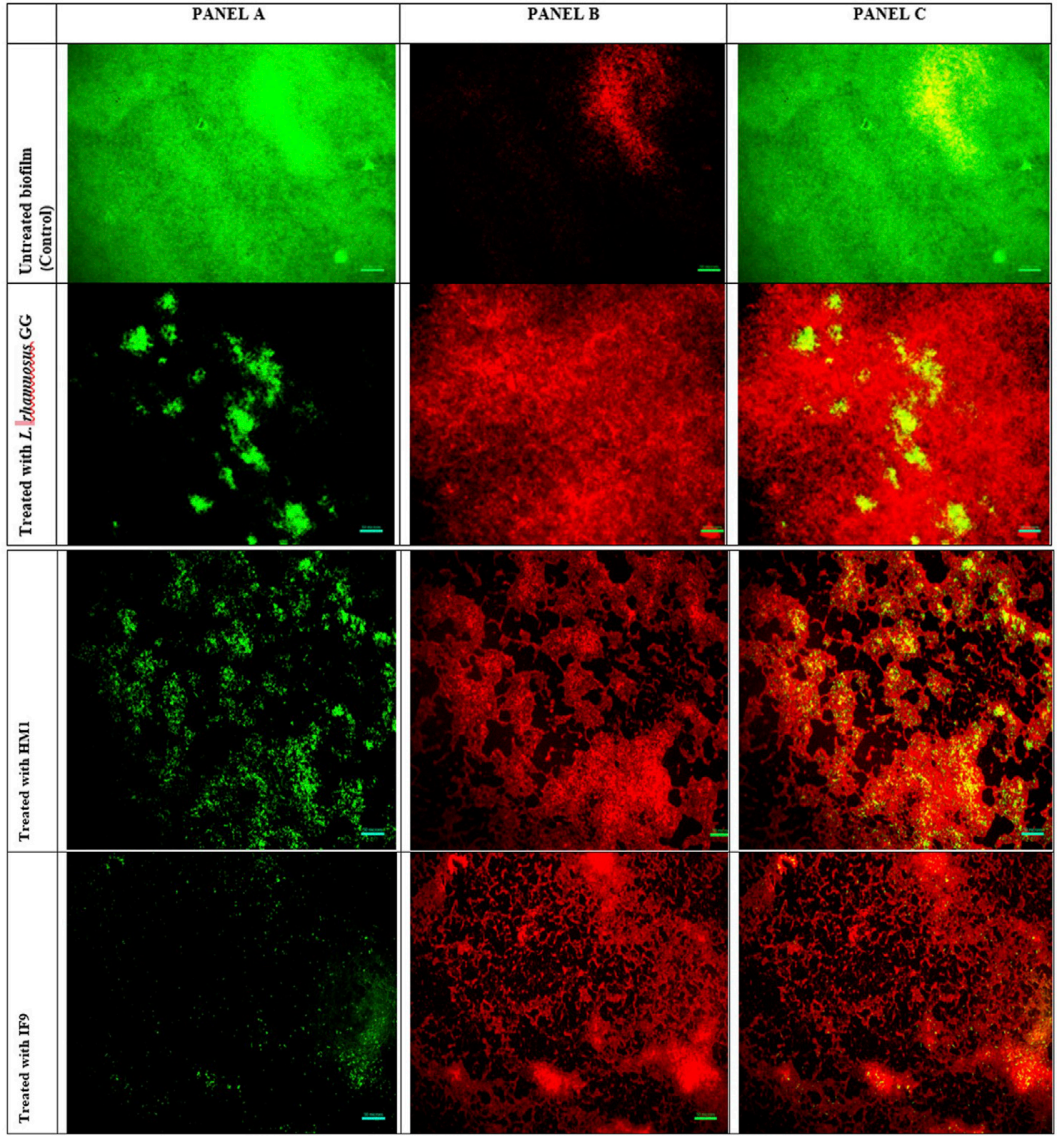
Fluorescent microscopic images of biofilms of *P. aeruginosa* grown in the absence and presence of un-treated cell free supernatant of *Lactobacillus*. **(A)** (left) is the image obtained from the green channel (live cells), **(B)** (center) from the red channel (dead cells) and **(C)** (right) is a merged image.

Visualization with light and fluorescent microscopy showed lower degrees of biofilm development in CFS-treated groups, as evidenced by a reduction in organized cell communities. In general, the biofilms presented distinct morphological changes with a poorly developed architecture upon the challenge with untreated CFS, compared to the control group (Figure 2). Following 48 h of incubation, the well-developed biofilm of test pathogens was observed in control wells; on the other hand, upon the CFS challenge, scattered cell growth was witnessed. Furthermore, fluorescent microscopic images also supported the anti-biofilm nature of LAB isolates, HM1 and IF9. Co-challenge with the untreated CFS of both the isolates disrupted the biofilm architecture compared to the control group (Figures 3, 4).

### 3.6 Principal component analysis

**FIGURE 5:**
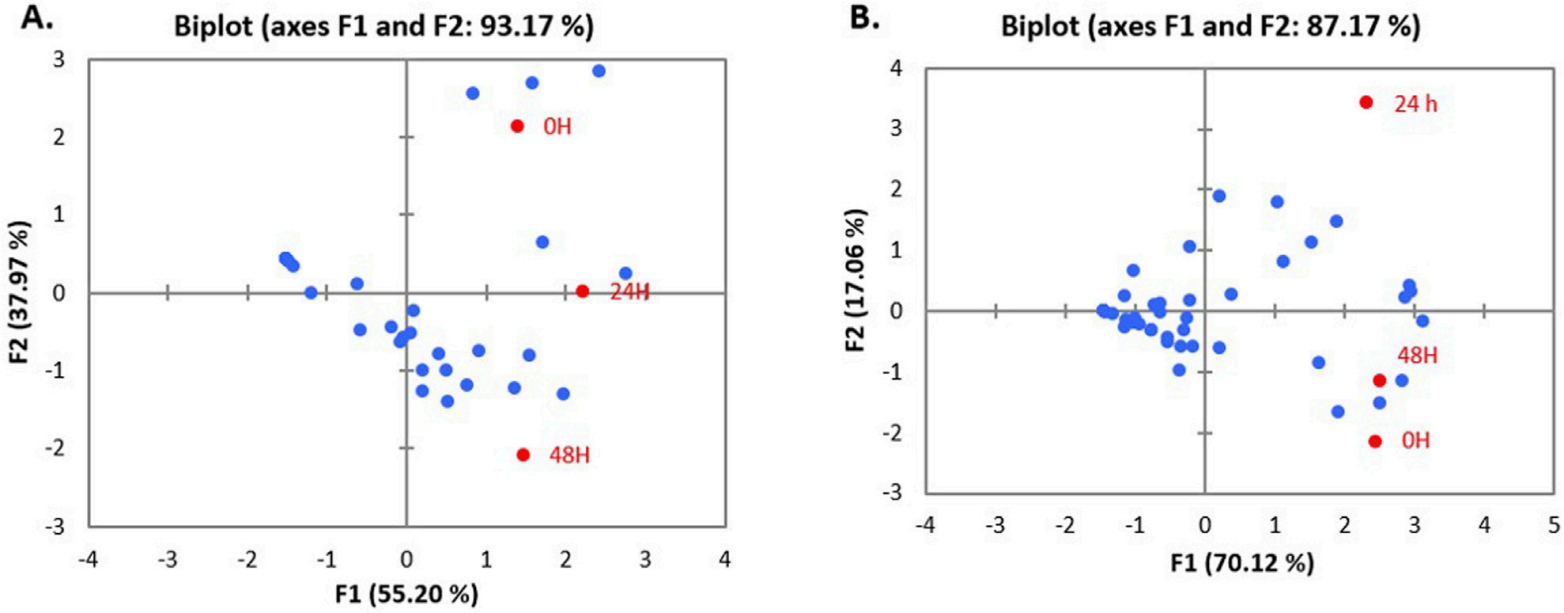
PCA biplot of treatment of un-treated CFS (a), neutralized CFS (b) and heat-treated CFS (c) of LAB isolated from human milk **(A)** and infant faecal **(B)** sample on the biofilm inhibition of *S. aureus*.

**FIGURE 6:**
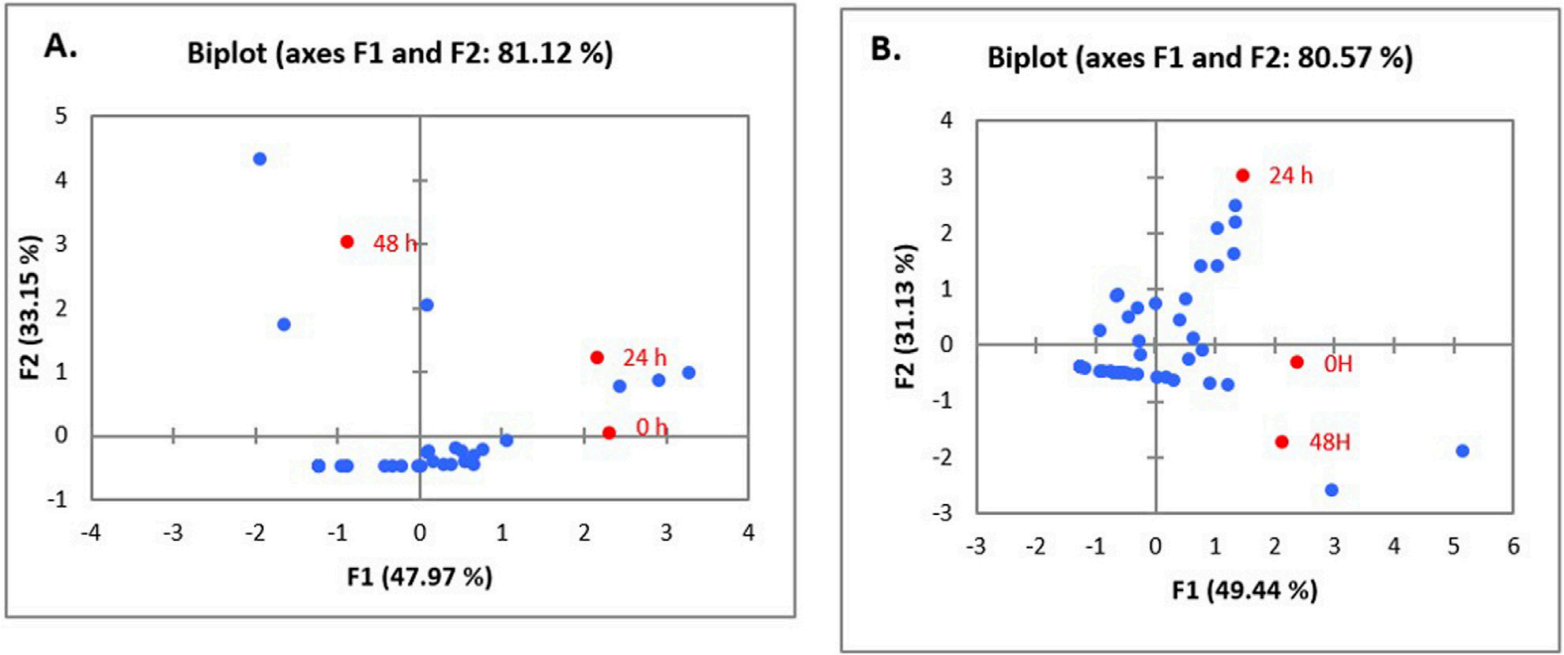
PCA biplot of treatment of un-treated CFS (a), neutralized CFS (b) and heat-treated CFS (c) of LAB isolated from human milk **(A)** and infant faecal **(B)** sample on the biofilm inhibition of *P. aeruginosa*.

**FIGURE 7:**
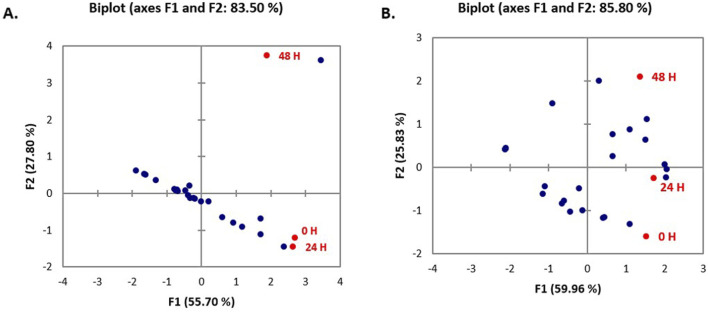
PCA biplot of un-treated CFS (a), neutralized CFS (b) and heat-treated CFS (c) of Lactobacillus isolates on the biofilm inhibition of *P. aeruginosa* **(A)** and *S. aureus* **(B)**.

Data emerging from biofilm inactivation experiments were subjected to statistical analysis using PCA (Supplementary Figures S1–S3). Figures 5, 6 document the PCA biplot, which characterizes the effect of untreated CFS (a), N-CFS (b), and H-CFS (c) of LAB isolates of breast milk and infant fecal origin on the biofilms of *S. aureus* and *P. aeruginosa*. On the basis of factor score values, untreated CFS of HM1, HM8, HM13, IF1, IF9, and IF11 most significantly impaired *S. aureus* biofilm under both treatment strategies (Supplementary Table S1). Under similar conditions, the *P. aeruginosa* biofilm was most significantly impaired by untreated CFS of HM1, HM2, HM11, IF13, IF14, and IF15 (Supplementary Table S2). Furthermore, the above mentioned isolates were shortlisted and subjected to normalized *PCA* with the reference probiotic *L. rhamnosus* GG and *L. casei* (Supplementary Table S3). Upon PCA normalization, *L. paracasei* HM1 emerged to be the most promising strain with biofilm inactivation values similar to or better than the reference probiotic strains against both pathogens (Figure 7).

## 4 Discussion

Biofilm-mediated bacterial infections are often very difficult to treat due to resistance conferred against antimicrobials and insensitivity to host immune response (Srinivasan et al., 2021). Biofilm formation by *S. aureus* and *P. aeruginosa* is a major concern in various sectors, posing challenges to public health and industry (Shineh et al., 2023; Wandhare et al., 2021). According to earlier research, probiotics possess characteristics that prevent *P. aeruginosa* from growing and developing its virulence components (Diaz et al., 2020). Strains of LAB and their metabolites have earlier been reported to prevent biofilm formation or even disrupt mature biofilms, indicating their potential application in the control of infections (Dincer, 2024; Al-Shamiri et al., 2023; Singh et al., 2018). The present study evaluated an array of LAB isolates from breast milk and infant fecal origin for their potential to retard biofilm formation or impair mature biofilms of two clinical isolates of *S*. *aureus* and *P. aeruginosa*. The overnight incubated untreated CFSs of test breast milk isolates have earlier been explored for their antibacterial effects against food spoilage and clinical pathogens. In one of our earlier published reports, no antagonistic activity of LAB CFS preparations was documented against *S. aureus*, while weak-to-average inhibition was recorded against *P. aeruginosa* (Sharma et al., 2017). The antimicrobial effects recorded with CFSs from LAB could be due to the metabolic activity-mediated reduction in pH by organic acids and the secretion of several bioactive molecules, including EPS, hydrogen peroxide, and biosurfactants with antimicrobial properties (Asadzadegan et al., 2023; Abou Elez et al., 2023). Interestingly, few of the isolates displayed poor antimicrobial activity but exhibited potent anti-biofilm activity, which may be due to the presence of surface-bound or secreted biosurfactants with anti-adhesive properties. Recent research has shown that the *Lactobacillus* probiotic strains reduce *P. aeruginosa’s* pathogenicity factors, such as protease, elastase, antibiofilm, and antipyocyanin (Asadzadegan et al., 2023; Chappel and Nair, 2020). Jeyanathan et al. (2021) also reported the antagonistic activity of cell-free preparations (from LAB) against planktonic cells, the initial adhesion phase, and the mature biofilm of *P. aeruginosa*.

Results emerging from antimicrobial, cell viability, and microscopic analyses establish the anti-biofilm potential of test LAB isolates. Among the LAB isolates, cell-free preparations of few breast milk and infant fecal isolates displayed the potent anti-biofilm effect against both pathogens. The effects were observed to be strain-specific as different strains belonging to the same species exerted variable degrees of supernatant-mediated biofilm inactivation. Concentrated metabolites and biosurfactants released in the spent media, along with competition for nutrients and interference with essential enzymes and gene expression, may have contributed to the inhibition of *S. aureus* and *P*. *aeruginosa* biofilms (Jeyanathan et al., 2021). The extent of anti-biofilm activity displayed by untreated CFSs was considerably reduced upon pH neutralization. Our results in this regard can be corroborated with an earlier study, where *S. aureus* biofilm inactivation by CFSs of *L. plantarum* and *L. rhamnosus* was notably reduced by approximately 63% (Carvalho et al., 2021). This suppressed activity indicates the role of low pH, induced by organic acids secreted during LAB metabolism. Previous research by Hu et al. (2019) identified several organic acids (lactic, tartaric, acetic, citric, and malic acids) in the CFSs of isolates of *L. plantarum*. Additionally, they demonstrated the antibacterial efficacy of these strains’ CFSs against *S. aureus*. Furthermore, the latest study showed that the CFSs of *L. casei* and *L. rhamnosus* dramatically decreased cell surface hydrophobicity, initial attachment, and biofilm formation and eradicated the biofilms (Saidi et al., 2023). Additionally, they demonstrated that the expression of the biofilm-forming gene (*sar*A) in *S. aureus* was decreased by the *L. casei* and *L. rhamnosus* CFSs. It is imperative to note that the *sar*A gene induces initial attachment and allows biofilm formation by suppressing the extracellular proteolytic and nucleolytic enzymes. As such, its downregulated expression inhibits *S. aureus* cells’ early adhesion. The effect of CFS of *L. paracasei* B31-2 on the production of biofilm by *Listeria monocytogenes* was also assessed in a recent work by Behbahani and colleagues. It was established that treatment with CFS of *L. paracasei* resulted in a reduction of initial formation and matured biofilm (Behbahani et al., 2024).

Recently, Chappel and Nair (2020) reported *P. aeruginosa* growth and biofilm formation inhibition by *L. plantarum* and *L. rhamnosus* GG. In another report, both acidic and neutralized LAB CFSs displayed anti-biofilm activity against *P. aeruginosa* PAO1 (Rana et al., 2020). On a similar note, *L. acidophilus*, *L. plantarum*, *L. johnsonii*, and *L. delbrueckii* CFS inhibited the biofilm formation and also dislodged the preformed biofilms of *P. aeruginosa* (Drumond et al., 2023). Lactobacilli preparations retained their anti-biofilm activity, following pH neutralization, indicating the involvement of other antimicrobial metabolites, such as antimicrobial peptides (AMPs), bacteriocins, and biosurfactants (Singh et al., 2018). Recently, Li et al. (2021) isolated and characterized a novel bacteriocin (*XJS01*) produced from the *L. salivarius* strain and established its antibacterial and anti-biofilm properties against *S. aureus.* The presence of sodium lactate, a neutralized form of lactic acid, or other low-molecular weight active substances may also be responsible for the anti-biofilm activity of neutralized preparations (Kaya et al., 2024; Kawai et al., 2016). Biosurfactants from LAB, such as proteinaceous components (Atanassova et al., 2003), hydrogen peroxide (H_2_O_2_) (Strus et al., 2005), cyclic dipeptides, and low-molecular weight compounds (Ryu et al., 2014), may reduce the surface substratum hydrophobicity and impair microbial adhesion and desorption (Saravanakumari and Mani, 2010). An earlier study by Shokouhfard et al. (2015) documented *S. marcescens* biofilm inactivation by *L. acidophilus* biosurfactants.

Heat treatment also influenced the anti-biofilm capability of CFSs to a varied degree. The activity was either lost, marginally reduced, or even increased for few of the isolates. Our results in this regard are in accordance with previous reports (Kiousi et al., 2023; Mariam et al., 2014), where the heat-stable biofilm inhibitory activity of LAB CFSs was documented. These reports indicate the non-proteinaceous nature of the active component within the CFS. In contrast, Ramos et al. (2015) reported the heat-sensitive anti-biofilm activity of *L. plantarum* CFS against *P. aeruginosa*. It is likely that the anti-biofilm potential resulted from a synergistic effect between low pH and the metabolites.

The CFSs demonstrated a limited ability to suppress pre-formed pathogen biofilms. The challenge with CFSs could efficiently retard early pathogen attachment and biofilm development (initial adhesion); however, they were unable to suppress/eliminate pre-formed biofilms to the same extent. This indicates that the effects on the biofilms might be physicochemical or, more particularly, interfacial in nature. In comparison to the control (test pathogens grown in BHI alone), no significant anti-biofilm activity was recorded with MRS and heat-treated MRS (H-MRS) under both co- and post-incubation challenges for both the test pathogens, as earlier reported by Mariam et al., (2014).

In this study, the impact of LAB CFSs on pathogen cell viability was also determined using the MTT assay. The cell-free preparations from reference probiotic strains *viz*. *L. rhamnosus* GG and *L. casei* along with test LAB isolates, HM1 and IF9, were found to appreciably reduce the levels of viable cells and sessile cells of *S. aureus* and *P. aeruginosa*, suggesting that the presence of LAB CFSs could not only inhibit the initial growth of pathogenic bacteria but also suppress biofilm formation. The declined number of viable cells of tested pathogens after exposure to CFSs could be due to metabolites present in the supernatant altering the architecture of pathogen’s biofilms by downregulating the genes involved in biofilm development and those associated with DNA replication, translation, glycolysis, and gluconeogenesis (Saidi et al., 2023; Chew et al., 2015). Earlier, the capability of other frequently used probiotic strains (*L. acidophilus* DSM 20079, *L. paracasei* DSMZ 16671, *L. plantarum* 299v, *L. rhamnosus* GG, and *L. reuteri* strains PTA 5289, etc.) to impede *Streptococcus mutans* growth and biofilm formation has been documented, and these data suggest that the antibacterial activity of *Lactobacillus* spp. appears to be strain-specific and pH-dependent (Rawal and Ali, 2023; Keller et al., 2011).

The microscopic analysis of the biofilms formed by *S. aureus* and *P. aeruginosa* in the presence or absence of untreated CFSs revealed a dramatic decrease in the adhesion of both the pathogen cells to the cover slips. Representative light microscopic and fluorescent images indicated the disruptive effects of CFSs from different LAB strains. Together, our data propose that the CFSs may affect biofilms by interfering with bacterial attachment and destabilizing the biofilm matrix. Candela et al. (2008) reported that different LAB strains could obstruct the adhesion sites for enteropathogens *E. coli* H10407 and *Salmonella typhimurium* on Caco-2 cells. In another study, the extracellular polysaccharides liberated from *L. acidophilus* A4 declined biofilms of enterohemorrhagic *E. coli* (EHEC) *in vitro* by affecting genes related to chemotaxis and curli production (Kim and Kim, 2009). They may also alter the biofilm integrity *via* interference with cell-to-cell aggregation and surface attachment processes. This effect may be mediated by the exo-polysaccharide released by LAB or by the physicochemical properties of their cell surface (Bernal and Llmas, 2012).

The data obtained different sets of experiments were statistically analyzed for categorizing the most prospective anti-biofilm strains of LAB. A data matrix was constructed, with samples organized in rows and response variables corresponding to different cell-free preparations, *viz*., untreated CFS (a), neutralized CFS (b), and heat-treated CFS (c), placed in columns. These preparations, derived from lactobacilli of breast milk and infant fecal origin, were evaluated for their biofilm-inactivating effects on *P. aeruginosa* and *S. aureus* with respect to different challenge strategies, and 2D plots were generated to predict the variability among the principal components. The first two components accounted for maximum variability (in the range of 80%–95%), which reflects that the outcome was generated without any information loss.

In conclusion, findings from this study validate the hypothesis that the therapeutic effectiveness of lactobacilli against foodborne and clinical pathogens is generally due to the interference with interfacial interactions (cell-to-cell and cell-to-surface) or due to the production of exo-metabolites that destabilize biofilm organization and architecture. The individual strains of LAB displayed strain-specific abilities to impair biofilm development by suppressing initial attachment. The shortlisted strains may hold promise for prophylactic and therapeutic applications against *S. aureus* and *P. aeruginosa*, which further need to be tested and validated through *in vivo* studies.

## Data Availability

The datasets presented in this study can be found in online repositories. The names of the repository/repositories and accession number(s) can be found in the article/Supplementary Material.
