# Supplementary material for: Exploring the antimicrobial potential of lactobacilli against early-stage and mature biofilms of Staphylococcus aureus and Pseudomonas aeruginosa
Source: Front Chem. 2025 Mar 21;13:1425666. doi: 10.3389/fchem.2025.1425666 (PMC11969340; doi:10.3389/fchem.2025.1425666)
Supplement: Supplementary file 1 [file DataSheet1.pdf]

Supplemental Files

Supplementary Figures

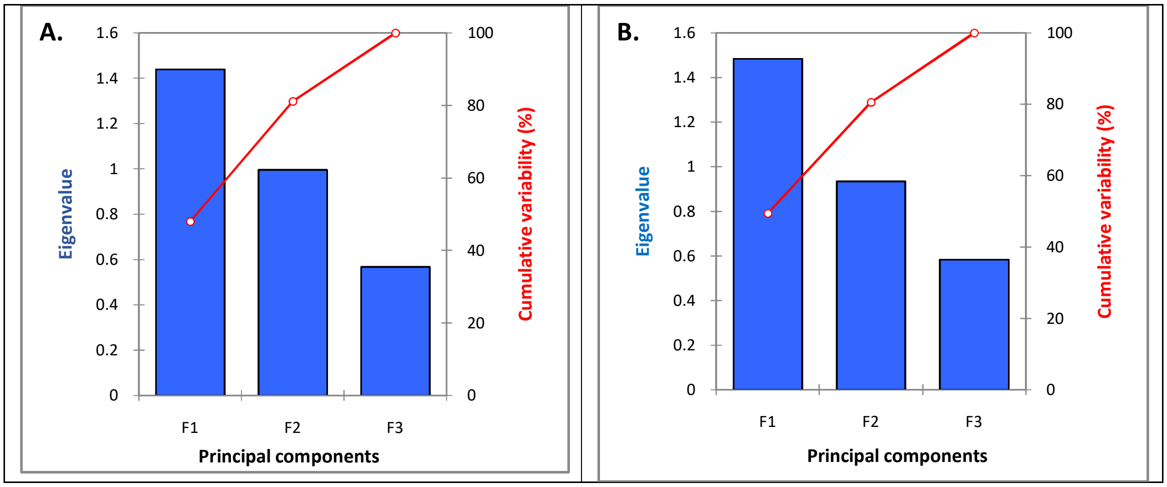

Supplementary Figure 1: Scree biplot (Eigenvalues) of principal components for treatment of un-treated CFS (a), neutralized CFS (b) and heat-treated CFS (c) of LAB isolated from human milk (A) and infant faecal (B) sample on the biofilm inhibition of *P. aeruginosa.*

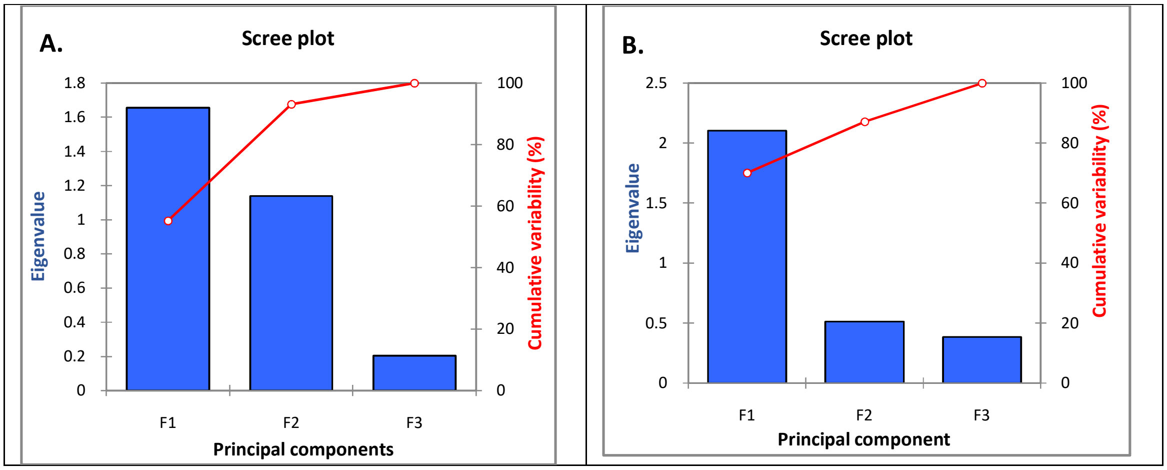

Supplementary Figure 2: Scree biplot (Eigenvalues) of principal components for treatment of un-treated CFS (a), neutralized CFS (b) and heat-treated CFS(c) of LAB isolated from human milk (A) and infant faecal (B) sample on the biofilm inhibition of *S. aureus.*

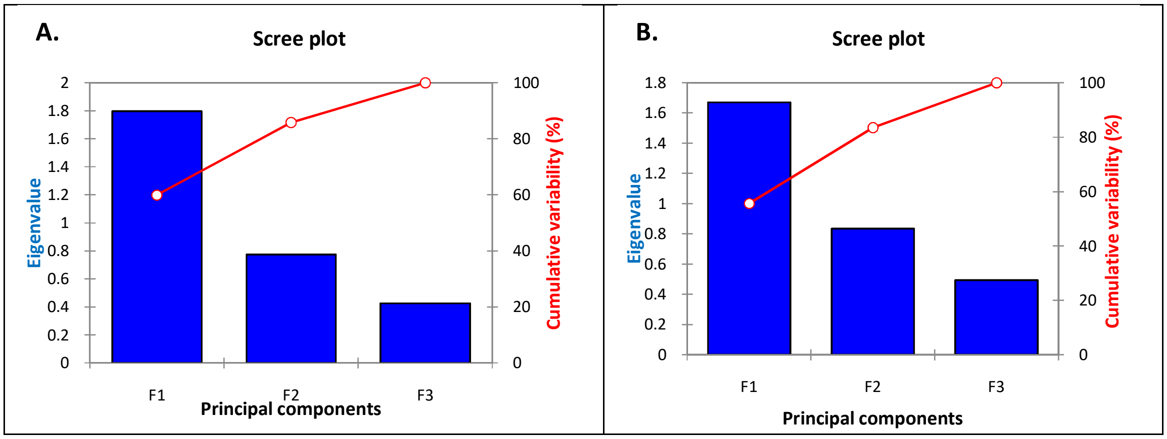

Supplementary Figure 3: Scree biplot (Eigenvalues) of principal components for un-treated CFS (a), neutralized CFS (b) and heat-treated CFS(c) of *Lactobacillus* isolates on the biofilm inhibition of *P. aeruginosa* (A) and *S. aureus* (B)

Supplementary Tables

**Supplementary Table 1**: **Factor score principal components for treatment of non-treated CFS (a), neutralized CFS (b) and heat-treated CFS (c) of LAB isolated from human milk (A) and infant faecal sample (B) on the biofilm inhibition of *Staphylococcus aureus***							
**Observation (A)**	**F1**	**F2**	**F3**	**Observation (B)**	**F1**	**F2**	**F3**
HM1a	2.422	2.850	0.560	IF1a	2.498	-1.504	0.345
HM1b	0.837	2.564	-0.523	IF1b	-1.466	0.007	-0.339
HM1c	1.576	2.699	-0.020	IF1c	1.917	-1.659	-0.397
HM2a	0.090	-0.217	0.600	IF2a	2.851	0.232	0.466
HM2b	-1.514	0.447	0.087	IF2b	-1.442	-0.003	-0.309
HM2c	-0.637	0.128	0.429	IF2c	1.628	-0.849	0.260
HM3a	0.508	-1.385	-0.651	IF3a	-0.314	-0.309	0.968
HM3b	-1.442	0.343	0.008	IF3b	-1.132	-0.144	0.088
HM3c	-0.202	-0.429	0.043	IF3c	-1.015	-0.088	0.149
HM6a	0.770	-1.196	-0.155	IF4a	-0.176	-0.577	1.309
HM6b	-1.514	0.447	0.087	IF4b	-0.538	-0.413	0.846
HM6c	-0.045	-0.569	-0.010	IF4c	0.185	-0.589	0.627
HM7a	0.212	-1.254	-0.734	IF5a	-1.333	-0.053	-0.170
HM7b	-1.514	0.447	0.087	IF5b	-1.454	0.002	-0.324
HM7c	-0.571	-0.464	-0.335	IF5c	-1.156	0.265	-0.261
HM8a	1.702	0.645	-0.915	IF6a	-0.652	-0.022	0.430
HM8b	-1.484	0.403	0.054	IF6b	-1.466	0.007	-0.339
HM8c	-0.097	-0.641	-0.157	IF6c	-0.780	-0.303	0.537
HM9a	0.194	-0.983	-0.888	IF7a	-0.348	-0.578	0.206
HM9b	-1.514	0.447	0.087	IF7b	-1.456	0.003	-0.326
HM9c	-1.208	0.011	-0.245	IF7c	-0.532	-0.504	0.349
HM10a	1.971	-1.296	0.782	IF8a	1.036	1.809	0.516
HM10b	-1.514	0.447	0.087	IF8b	-1.165	-0.255	-0.668
HM10c	0.399	-0.779	0.095	IF8c	-0.220	0.187	-0.040
HM11a	0.499	-0.977	-0.092	IF9a	2.825	-1.136	0.054
HM11b	-1.514	0.447	0.087	IF9b	-0.376	-0.942	-1.529
HM11c	0.052	-0.515	0.152	IF9c	3.103	-0.153	-0.820
HM12a	1.538	-0.802	1.081	IF10a	-0.769	-0.308	0.551

HM12b	-1.514	0.447	0.087	IF10b	-0.963	-0.220	0.303
HM12c	0.896	-0.746	0.585	IF10c	-0.275	-0.089	-0.344
HM13a	2.766	0.270	-0.687	IF11a	2.961	0.353	-0.690
HM13b	-1.498	0.423	0.069	IF11b	-1.457	0.003	-0.328
HM13c	1.352	-1.213	0.344	IF11c	2.934	0.426	-1.062
				IF12a	1.883	1.497	1.541
				IF12b	-1.035	-0.188	0.211
				IF12c	0.201	1.891	-0.315
				IF13a	1.121	0.817	-1.316
				IF13b	-1.466	0.007	-0.339
				IF13c	-0.223	1.063	-0.042
				IF14a	1.532	1.137	0.087
				IF14b	-1.466	0.007	-0.339
				IF14c	-0.710	0.103	-0.327
				IF15a	0.373	0.282	0.913
				IF15b	-1.023	0.660	-0.455
				IF15c	-0.640	0.124	0.325

Supplementary Table 2: **Factor score of principal components for treatment of un-treated CFS (a), neutralized CFS (b) and heat-treated CFS(c) of LAB isolated from human milk (A) and infant faecal sample (B) on the biofilm inhibition of *Pseudomonas aeruginosa***

Observation (A)	F1	F2	F3	Observation (B)	F1	F2	F3
HM1a	3.268	0.985	-1.224	IF1a	0.021	-0.568	0.809
HM1b	2.890	0.855	-1.159	IF1b	0.767	1.421	-0.035
HM1c	2.435	0.768	-1.334	IF1c	-0.243	-0.146	0.219
HM2a	0.085	2.053	2.000	IF2a	1.021	1.412	0.219
HM2b	-0.917	-0.473	-0.441	IF2b	-0.758	-0.468	-0.028
HM2c	0.371	-0.449	0.856	IF2c	-0.681	-0.478	0.055
HM3a	0.006	-0.456	0.488	IF3a	0.397	0.446	0.374
HM3b	-1.241	-0.479	-0.767	IF3b	-1.261	-0.403	-0.569
HM3c	-0.884	-0.472	-0.408	IF3c	-0.952	-0.443	-0.236
HM6a	0.758	-0.223	0.451	IF4a	-0.310	0.684	-0.501
HM6b	-1.241	-0.479	-0.767	IF4b	-0.456	0.528	-0.521
HM6c	-0.231	-0.460	0.250	IF4c	-1.261	-0.403	-0.569
HM7a	0.081	-0.279	-0.072	IF5a	0.540	-0.256	1.069
HM7b	-1.241	-0.479	-0.767	IF5b	-1.261	-0.403	-0.569
HM7c	-0.430	-0.464	0.050	IF5c	-0.571	-0.492	0.173
HM8a	0.659	-0.309	0.660	IF6a	1.346	2.178	-0.068
HM8b	-1.241	-0.479	-0.767	IF6b	-0.941	0.267	-0.787
HM8c	-1.241	-0.479	-0.767	IF6c	-1.261	-0.403	-0.569
HM9a	0.141	-0.407	0.455	IF7a	0.503	0.847	0.162
HM9b	-1.241	-0.479	-0.767	IF7b	-0.429	-0.509	0.325
HM9c	-0.951	-0.474	-0.474	IF7c	-1.261	-0.403	-0.569
HM10a	0.546	-0.417	0.928	IF8a	0.629	0.137	0.845
HM10b	0.108	-0.240	-0.185	IF8b	-0.658	0.859	-0.979
HM10c	-0.033	-0.456	0.449	IF8c	-0.278	0.055	0.027
HM11a	1.044	-0.063	0.182	IF9a	1.300	1.657	0.298
HM11b	0.425	-0.184	-0.045	IF9b	-0.623	-0.485	0.117
HM11c	0.503	-0.240	0.242	IF9c	-0.605	-0.488	0.136
HM12a	0.583	-0.418	0.972	IF10a	1.338	2.469	-0.306
HM12b	-1.665	1.726	0.071	IF10b	-0.630	0.918	-0.998
HM12c	-0.335	-0.462	0.145	IF10c	-0.012	0.730	-0.248

HM13a	0.276	-0.451	0.760	IF11a	1.043	2.089	-0.294
HM13b	-1.942	4.328	-0.160	IF11b	-1.261	-0.403	-0.569
HM13c	0.655	-0.444	1.141	IF11c	-0.558	-0.494	0.187
				IF12a	0.181	-0.589	0.982
				IF12b	-1.189	-0.413	-0.491
				IF12c	-0.295	-0.527	0.470
				IF13a	5.147	-1.876	-2.165
				IF13b	0.805	-0.104	1.206
				IF13c	-0.521	-0.498	0.226
				IF14a	2.955	-2.578	-1.067
				IF14b	0.301	-0.604	1.111
				IF14c	0.915	-0.683	1.771
				IF15a	1.205	-0.720	2.083
				IF15b	-0.879	-0.452	-0.158
				IF15c	-1.261	-0.403	-0.569

Supplementary Table 3: **Factor score of principal components for treatment un-treated CFS (a), neutralized CFS (b) and heat-treated CFS(c) of LAB isolated from human milk and infant faecal sample on the biofilm inhibition of *Pseudomonas aeruginosa* (A) and *Staphylococcus aureus* (B).**

Observation (A)	F1	F2	F3	Observation (B)	F1	F2	F3
***L. rhamnosus* GG a**	2.3733	-1.4455	0.2702	***L. rhamnosus* GG a**	0.3988	-1.1549	0.3406
***L. rhamnosus* GG b**	1.7193	-0.6847	-0.2663	***L. rhamnosus* GG b**	-0.2306	-0.4760	0.9935
***L. rhamnosus* GG c**	-0.1922	-0.1331	0.9799	***L. rhamnosus* GGc**	-0.6027	-0.7602	-0.2427
***L. casei* a**	1.7157	-1.1116	0.3980	***L. casei* a**	-0.4474	-1.0338	-0.6548
***L. casei* b**	3.4446	3.6149	-0.1323	***L. casei* b**	-0.6636	-0.8294	-0.4988
***L. casei* c**	-0.7431	0.1135	0.3607	***L. casei* c**	-1.0985	-0.4293	-0.2109
**HM1a**	1.1717	-0.9136	-1.2068	**HM1a**	1.0913	-1.3096	1.0236
**HM1b**	0.9152	-0.7836	-1.1635	**HM1b**	-0.1462	-0.9875	-0.0891
**HM1c**	0.6040	-0.6347	-1.3028	**HM1c**	0.4309	-1.1387	0.4276
**HM2a**	-0.3545	0.2138	0.6615	**HM8a**	0.6524	0.7624	-0.1342
**HM2b**	-1.6683	0.5275	-0.6790	**HM8b**	-2.1076	0.4624	0.1978
**HM2c**	-0.7790	0.1296	0.3204	**HM8c**	-0.9016	1.4987	0.3146
**HM11a**	-0.3276	-0.1185	-0.1781	**HM13a**	1.5365	1.1223	0.2448
**HM11b**	-0.7510	0.0845	-0.3594	**HM13b**	-2.1209	0.4416	0.2066
**HM11c**	-0.6957	0.0669	-0.1416	**HM13c**	0.2873	1.9986	0.9915
**IF13a**	0.2024	-0.2185	0.6216	**IF1a**	1.1025	0.8761	-1.2906
**IF13b**	-0.3994	-0.0517	0.4975	**IF1b**	-2.1370	0.4165	0.2172
**IF13c**	-1.3283	0.3754	-0.2968	**IF1c**	0.6459	0.2652	-1.4476
**IF14a**	-0.4671	0.0777	0.6274	**IF9a**	1.5057	0.6447	-0.9397
**IF14b**	-0.7014	0.0948	0.4076	**IF9b**	-1.1585	-0.6144	-0.7399
**IF14c**	-0.2334	-	0.9335	**IF9c**	2.0552	-0.0437	0.0053

		0.1146					
**IF15a**	-0.0123	-<br>0.2136	1.1819	**IF11a**	2.0003	0.0838	0.5029
**IF15b**	-1.6010	0.4974	-0.6033	**IF11b**	-2.1311	0.4258	0.2133
**IF15c**	-1.8919	0.6276	-0.9303	**IF11c**	2.0388	-0.2207	0.5690
